# AI-Driven Information for Relatives of Patients with Malignant Middle Cerebral Artery Infarction: A Preliminary Validation Study Using GPT-4o

**DOI:** 10.3390/brainsci15040391

**Published:** 2025-04-11

**Authors:** Mejdeddine Al Barajraji, Sami Barrit, Nawfel Ben-Hamouda, Ethan Harel, Nathan Torcida, Beatrice Pizzarotti, Nicolas Massager, Jerome R. Lechien

**Affiliations:** 1 Department of Neurosurgery, University Hospital of Lausanne and University of Lausanne, 1005 Lausanne, Switzerland; ethan.harel@hotmail.com; 2 Department of Neurosurgery, CHU Tivoli, 7110 La Louvière, Belgium; samibarrit@gmail.com (S.B.); nicolas.massager@ulb.be (N.M.); 3 Department of Adult Intensive Care, University Hospital of Lausanne (CHUV), University of Lausanne, 1005 Lausanne, Switzerland; nawfel.ben-hamouda@chuv.ch; 4 Department of Neurology, Hôpital Universitaire de Bruxelles (HUB), 1070 Brussels, Belgium; nathan.torcidasedano@hubruxelles.be; 5 Department of Neurology, University Hospital of Lausanne (CHUV), University of Lausanne, 1011 Lausanne, Switzerland; beatrice.pizzarotti@chuv.ch; 6 Department of Surgery, UMONS Research Institute for Health Sciences and Technology, University of Mons (UMons), 7000 Mons, Belgium; jerome.lechien@umons.ac.be; 7 Department of Otolaryngology, Elsan Polyclinic of Poitiers, 86000 Poitiers, France; 8 Department of Otolaryngology-Head Neck Surgery, Foch Hospital, School of Medicine, UFR Simone Veil, Université Versailles Saint-Quentin-en-Yvelines (Paris Saclay University), 78035 Paris, France

**Keywords:** artificial intelligence, ChatGPT, decompressive hemicraniectomy, stroke

## Abstract

**Purpose:** This study examines GPT-4o’s ability to communicate effectively with relatives of patients undergoing decompressive hemicraniectomy (DHC) after malignant middle cerebral artery infarction (MMCAI). **Methods:** GPT-4o was asked 25 common questions from patients’ relatives about DHC for MMCAI, twice over a 7-day interval. Responses were rated for accuracy, clarity, relevance, completeness, sourcing, and usefulness by board-certified intensivist* (one), neurologists, and neurosurgeons using the Quality Analysis of Medical AI (QAMAI) tool. Interrater reliability and stability were measured using ICC and Pearson’s correlation. **Results:** The total QAMAI scores were 22.32 ± 3.08 for the intensivist, 24.68 ± 2.8 for the neurologist, 23.36 ± 2.86 and 26.32 ± 2.91 for the neurosurgeons, representing moderate-to-high accuracy. The evaluators reported moderate ICC (0.631, 95% CI: 0.321–0.821). The highest subscores were for the categories of accuracy, clarity, and relevance while the poorest were associated with completeness, usefulness, and sourcing. GPT-4o did not systematically provide references for their responses. The stability analysis reported moderate-to-high stability. The readability assessment revealed an FRE score of 7.23, an FKG score of 15.87 and a GF index of 18.15. **Conclusions:** GPT-4o provides moderate-to-high quality information related to DHC for MMCAI, with strengths in accuracy, clarity, and relevance. However, limitations in completeness, sourcing, and readability may impact its effectiveness in patient or their relatives’ education.

## 1. Introduction

The development of large language models (LLMs) has gained considerable ground in medicine. ChatGPT, based on OpenAI’s series of generative pre-trained transformer (GPT) models, is among the most popular LLM-driven chatbots used by both patients and practitioners. This increasing utilization has spurred numerous studies aimed at evaluating the quality of the medical information provided by such models [1,2]. LLM-based applications have been examined across a wide array of medical specialties, including neurology [3,4], cardiology [5,6], infectious diseases [7,8], oncology [9,10,11,12], hematology [13], gastroenterology [14,15], urology [11,16,17], gynecology and obstetrics [18,19], and emergency medicine [20] as well as surgical disciplines such as neurosurgery [21] and head and neck surgery [22,23]. While substantial research has explored the use of LLM in various healthcare contexts—including medical education, clinical practice, research, and ethical considerations—there remains a critical gap in the literature concerning their performance in providing accessible medical information to laypersons, particularly patients and their relatives. To our knowledge, no studies have specifically addressed the role of ChatGPT or similar public LLM-based solutions in assisting relatives of critically ill patients to understand medical information in intensive care settings. This gap is especially pertinent in critical care, where high-stakes treatments and life-or-death decisions often occur in the wake of complex diagnoses. In such emotionally charged and cognitively overwhelming situations, relatives are confronted with a barrage of complex medical information, which they may struggle to fully comprehend. Consequently, they might seek supplemental information from widely available and increasingly popular sources, including chatbot-based platforms [24,25]. A particularly illustrative example is malignant middle cerebral artery infarction (MMCAI), a severe condition associated with brain injury and edema, which frequently necessitates decompressive hemicraniectomy (DHC) as a life-saving intervention in neurocritical care [26].

This study seeks to evaluate the capacity of a state-of-the-art LLM to address common questions posed by relatives about DHC and MMCAI, delineating its potential to support families during critical care episodes.

## 2. Methods

### 2.1. Questions and Setting

GPT-4o (OpenAI, San Francisco, CA, USA) was tasked with providing explanations in hypothetical conversations with relatives of patients diagnosed with MMCAI and candidates for DHC. Twenty-five questions commonly asked by patients’ relatives regarding DHC in case of an MMCAI were collected by 7 practitioners, including a board-certified intensivist (refs. [1]), two neurologists (refs. [2,3]) and three neurosurgeons (refs. [4,5,6]). The questions covered specific subtopics: indication (N = 3); surgical procedure (N = 3); postoperative care (N = 7); prognosis (N = 4); outcomes (N = 4); ethical issues (N = 1); and rehabilitation (N = 3). All questions were independently submitted twice, seven days apart, into the GPT-4o web application interface (https://chat.openai.com, accessed on 8 August 2024). The complete set of questions is available in Table 1. The generated responses were compiled into a document provided to four evaluators (refs. [1,2,5,6]). See Supplementary Files S1 and S2.

### 2.2. Quality Analysis

**Figure 1:**
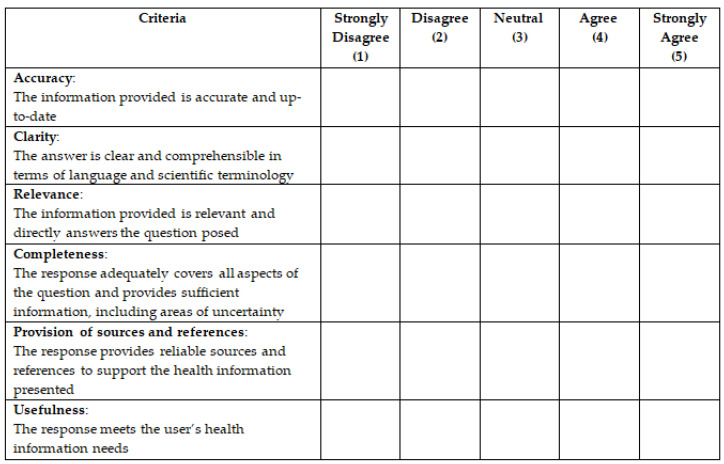
The Quality Analysis of Medical Artificial Intelligence tool. Each item is assessed using a 5-point Likert scale (strongly disagree, disagree, neutral, agree, strongly agree).

The study’s endpoints evaluated the accuracy, clarity, relevance, completeness, sourcing, and usefulness of the answers, as independently reviewed by the multidisciplinary team previously introduced, using the Quality Analysis of Medical Artificial Intelligence (QAMAI) tool (Figure 1).

The QAMAI tool [27] is a validated and standardized instrument specifically designed to assess the quality of health information provided by AI chatbots. This tool is inspired by the modified DISCERN [28] instrument (mDISCERN), a robust and widely adopted tool for monitoring the quality of health information on websites, social media, and related platforms. Each mDISCERN parameter is rated on a 5-point Likert scale ranging from 1 (strongly disagree) to 5 (strongly agree). These ratings are summed to form an aggregate score (QAMAI score), which reflects the overall quality of the provided information. Additionally, the readability of the answers was assessed using the Flesch Reading Ease (FRE) score, the Flesch–Kincaid Grade (FKG), and the Gunning Fog Index (GFI) [29].

### 2.3. Statistical Methods

Statistical analyses were conducted with the Statistical Package for the Social Sciences for Windows (SPSS version 30.0; IBM Corp., Armonk, NY, USA). Accuracy, clarity, relevance, completeness, reference, usefulness, and total QAMAI scores of GPT-4o answers were all reported with means and standard deviations. Inter-rater reliability was evaluated using the intraclass correlation coefficient (ICC). The stability of GPT-4o answers was tested using Pearson’s correlation coefficient and was categorized as low (k < 0.40), moderate (0.40–0.60), or strong (k > 0.60). A significance level of *p* < 0.05 was applied.

## 3. Results

The QAMAI scores of the answers provided by GPT-4o are presented in Table 2. Total QAMAI scores were 22.32 ± 3.08 for the intensivist, 24.68 ± 2.8 for the neurologist, 23.36 ± 2.86 and 26.32 ± 2.91 for the neurosurgeons (*p* = 0.120), demonstrating moderate-to-high quality information. GPT-4o scored the highest in accuracy (mean: 4.40/5, *p* < 0.001), clarity (mean: 4.53/5, *p* < 0.001), and relevance of explanation (mean: 4.51/5, *p* < 0.001), particularly for Ethical Issues and Rehabilitation subtopics. The lower subscores were associated with completeness (mean: 4.02/5, *p* < 0.001), usefulness (mean: 4.26/5, *p* < 0.001), and information sourcing, consistently scoring the poorest (mean: 2.85/5, *p* < 0.001), particularly in postoperative care and prognosis subtopics.

The analysis of the GPT-4o answers, detailed in Table 3, shows moderate to strong stability for all answers. Inter-rater reliability assessment suggested substantial agreement, with an ICC of 0.631 (95% CI: 0.321–0.821). The readability assessment of the answers revealed an FRE score of 7.23, an FKG score of 15.87, and a GFI of 18.15, appropriate for a graduate or postgraduate level of education.

## 4. Discussion

Stroke remains a leading cause of death and disability worldwide, with 13.7 million new cases and approximately 5.5 million related deaths reported annually [30]. Of these, up to 10% are MMCAI, a condition often complicated by severe mass-effect edema [31]. Untreated mortality rates may increase to 80%, primarily due to severe intracranial hypertension [32,33]. In this context, decompressive surgery, particularly DHC, has emerged as a key intervention [26,31,34].

In this study, GPT-4o exhibited moderate-to-high levels of accuracy, clarity, and relevance, aligning with reported findings on basic and specialized medical queries [1,35,36]. In the literature, accuracy rates range from 36% to 90% [1]. However, these findings should be interpreted cautiously due to the lack of standardized guidelines and validated benchmarks for assessing the performance of LLM, which results in inconsistent evaluations across studies [1]. Studies investigating accuracy using a Likert scale have reported ratings exceeding 80% for both GPT-3.5 and 4 [1,22,35], supporting that these models have significant potential for use in medical education and decision-making support. Accuracy remains consistent for binary and descriptive questions but declines with increasing contextual complexity, particularly in surgical scenarios [1,35]. This may stem from the procedural nature of surgery, which is difficult to convey through text-based interactions [1,2]. Similarly, advanced queries often require experiential and cultural knowledge that humans intuitively grasp through both experience and non-verbal communication but which may not be captured in the information explicitly provided to the model. As a result, LLM may struggle with nuanced real-world contexts despite proficiency in handling vast, detailed information. While these models can synthesize data effectively, they struggle with evolving data and the procedural expertise that healthcare professionals develop through hands-on experience and clinical training [37]. Finally, LLMs are limited by the cutoff date of their training data, meaning they lack access to the most recent medical literature and databases after that point. For instance, GPT-4o has been trained on information available up to October 2023, limiting its ability to provide up-to-date medical guidance. To address these limitations, advanced prompt engineering techniques but also fine-tuning, retrieval-augmented generation (RAG), and tailored user interfaces can be employed to specialize LLM for context-specific applications [38]. For instance, OpenAI recently introduced GPT-o1, a model designed to enhance efficiency in high-order reasoning tasks through native integration of chain-of-thought prompting [39,40]. Moreover, some LLM-driven solutions now offer dynamic access through web browsing and integration with personal documents via dedicated user interfaces—although the backend processes, including real-time retrieval architectures and grounding mechanisms, remain largely opaque and underexplored. Evaluating the performance of these approaches in similar applications could provide valuable insights into its potential improvements over GPT-4o.

Along these lines, GPT-4o did not systematically provide references, preventing the users from verifying the validity of every answer. This limitation underscores a core issue with GPT’s performance in sourcing information, as it often encounters difficulties in this area, sometimes producing erroneous or fabricated references [41,42,43]. For instance, Mishra et al. [44] examined GPT’s responses to queries on 40 common neurosurgical conditions. They found that while the overall quality of the information was fair, 69% of the references were inaccurate, with 34% being entirely fabricated. This issue is consistent with findings from Vaira et al. [45], who reported a 50% rate of false references in answers to head and neck surgery questions. These concerns had already been raised by Frosolini et al. [46] regarding GPT-3.5 and seem to persist in GPT-4o. In this context, RAG also offers a promising approach for grounding LLM-generated information by integrating real-time, verifiable sources into responses [47].

In line with our findings, previous studies [48,49,50,51] evaluating the readability of ChatGPT’s medical responses have shown that while the AI can generate high-quality and detailed information, it often fails to meet the recommended 6th-grade reading level typically advised for patient education [52]. Instead, ChatGPT’s medical explanations are frequently written at a college graduate level, limiting their accessibility and making it difficult for the general population to fully understand the content. It has been demonstrated that simplifying patient education materials significantly improves patient comprehension [53]. One study found that when AI was prompted to lower the grade level of its responses, the readability of the content improved considerably [54]. Despite the accuracy and depth of the information ChatGPT provides, the complexity of its language may hinder its effectiveness in educating patients.

The analysis of GPT-4o’s responses across two sessions demonstrated moderate to strong stability across most dimensions, with total score correlations indicating strong consistency. Such consistency is crucial for maintaining the quality, reliability, and reproducibility of the information provided [55]. This finding aligns with the over 90% reproducibility rates reported in the literature [56,57,58]. Nonetheless, Ashrafi et al. [59], in a study involving a high volume of repeated queries (741 questions, repeated 15 times), identified a tendency for ChatGPT to repeat specific errors or sporadically provide incorrect responses. While this level of repetition exceeds typical user interactions, it should be considered in decision-making contexts. This stochastic behavior is inherent to the architecture of LLM and is compounded by the proprietary and opaque nature of OpenAI’s models and infrastructure.

## 5. Limitations

The first limitation of this study is the moderate ICC value (0.631), which remains within an acceptable range according to the existing literature [60]. This moderate value may be attributed to the small sample size with substantial variability in responses due to discipline-specific perspectives. To improve the robustness of future studies, a larger and more diverse group of raters with varying expertise levels should be considered. A further limitation is the relatively small number of questions (n = 25) used to assess GPT-4o’s performance. While these were carefully selected to reflect common concerns, this limited sample may not fully capture the breadth and variability of questions posed by relatives in real-world settings. Future studies should consider including a larger, more randomized or crowd-sourced set of questions for a more comprehensive evaluation. Another limitation is the absence of relatives from the evaluation process in this preliminary study, precluding an assessment of the clarity of GPT-4o’s responses from the perspective of non-professional caregivers. While healthcare professionals assessed clarity, the best judges of how well GPT-4o informs patients and their relatives are, of course, the patients and relatives themselves. Their perspectives would offer the most direct and meaningful insights into the model’s effectiveness in real-world, patient-facing scenarios. This could be implemented as part of a two-phase study with this first phase involving the expert validation of medical content and the second phase incorporating feedback from patients’ relatives. Finally, our stability evaluation, based on repeating questions twice with a one-week interval, is insufficient to fully assess the consistency of responses from a closed-source, proprietary model such as GPT-4o. The model’s outputs may be influenced not only by its architecture but also its underlying infrastructure with dynamic back-end processes, including human-in-the-loop, customized training and pre/post-processing in/output adjustments, which are opaque in such proprietary models.

## 6. Perspectives

The future of LLM-driven applications in critical care public education hinges on developing accessible, transparent, and ethically sound solutions. Prioritizing open-source development will enable the creation of systems that are monitorable and auditable by healthcare professionals, while being tailored to specific populations and conditions. These systems should integrate advanced retrieval-augmented generation (RAG) techniques to provide verifiable, up-to-date, and curated information, addressing current sourcing limitations. Intuitive user interfaces will facilitate effective human–AI interaction, ensuring that AI enhances rather than replaces clinical expertise, which is critical for safety and ethical alignment. By improving readability and incorporating explainable AI principles, these solutions will promote transparency and usability for both clinicians and patients, fostering AI literacy in healthcare. Open-source, transparent LLM systems will also support thorough validation and ethical scrutiny through prospective studies, incorporating rigorous patient consent and data privacy measures.

From an ethical and legal standpoint, the potential impact of incomplete or inaccurate AI-generated information on the decision-making of patients’ relatives must be carefully considered. In high-stakes critical care contexts, families may rely heavily on readily accessible explanations to guide their understanding and expectations. If not adequately contextualized, such information could inadvertently shape consent and influence care-related decisions. To address this risk, the future implementations of LLMs should incorporate safeguards such as explicit disclaimers, prompts encouraging users to verify content with medical professionals, and transparency features including source citations or confidence scores. These measures are essential to ensuring that AI tools function as supportive educational aids rather than substitutes for clinical judgment, thereby reducing the risk of misinformation and promoting ethically responsible use in patient-facing communication.

Involving patients and families in development, alongside comprehensive trials, will ensure that LLM-driven tools are not only technically proficient but also aligned with real-world needs and ethical standards. This integrated approach addresses the key limitations identified in our study and paves the way for responsible, effective AI implementation in critical care education [61].

## 7. Conclusions

This study provides an analysis of GPT-4o’s ability to inform relatives about DHC for MMCAI. Our findings indicate that GPT-4o delivers moderate-to-high-quality information with relative stability, particularly in accuracy, clarity, and relevance. However, limitations in completeness, sourcing, and readability may hinder its effectiveness in public education. Studies involving all stakeholders, including larger multidisciplinary expert groups, are essential for robust and operable conclusions while evaluating LLM performance across various critical care conditions is crucial for generalizability. As LLM-driven solutions evolve, their role in medical communication must be carefully examined through open-source, transparent development and rigorous validation studies. Future efforts should prioritize purpose-built systems with improved readability, verifiable information sourcing, and intuitive interfaces, all underpinned by strong ethical considerations and real-world clinical validation.

## Figures and Tables

**Table 1:** Questions frequently asked by relatives of decompressive hemicraniectomy patients for malignant MCA infarct.

Questions
Indications:
1. What is a decompressive hemicraniectomy and why is it necessary in this case?
2. Are there any alternative treatments to decompressive hemicraniectomy for this condition?
3. Can the condition worsen if surgery is delayed, or do we have time to think about it?
Surgical Procedure:
4. How long will the surgery take?
5. What are the possible risks and complications associated with this surgery?
6. What happens to the brain without the skull to protect it?
Postoperative Care:
7. After surgery, when will relatives be allowed to see the patient?
8. How soon will the patient wake up?
9. During the coma period, can the patient hear me and how should I talk to them?
10. How long will the patient need to stay in the ICU and hospital after the surgery?
11. What type of care and support will be needed at home?
12. Will the patient need permanent assistance?
13. When will the removed part of the skull be replaced?
Prognosis:
14. What are the chances of survival?
15. What is the functional prognosis?
16. What are the chances of a full recovery?
17. What factors influence the patient’s recovery?
Outcomes:
18. How long will it take to see the maximum improvements in the patient’s condition?
19. What is the long-term impact on the patient’s cognitive abilities?
20. Will the patient be able to recognize his relatives?
21. Are there any aids to daily living that will be needed?
Ethical Issues:
22. What are the ethical considerations for withdrawing life support if necessary?
Rehabilitation:
23. What does rehabilitation consist of and how long will it take?
24. How can family members support the patient’s rehabilitation at home?
25. Are there any new or emerging rehabilitation techniques that could benefit the patient?

Abbreviations: ICU = intensive care unit.

**Table 2:** Quality Analysis of Medical Artificial Intelligence Scores.

	**Intensivist** [1]							
**QAMAI items** **(5-Likert Scale)**	**Indications** **(n = 3)**	**Surgical Procedure** **(n = 3)**	**Postoperative Care** **(n = 7)**	**Prognosis** **(n = 4)**	**Outcomes** **(n = 4)**	**Ethical Issues** **(n = 1)**	**Rehabilitation** **(n = 3)**	**Total** **(n = 25)**
Accuracy	3.67 ± 2.31	4.67 ± 0.58	4.14 ± 0.69	4.5 ± 0.58	4.5 ± 0.58	5	4.67 ± 0.58	4.36 ± 0.91
Clarity	5	3.67 ± 0.58	4.29 ± 0.76	3.75 ± 0.5	4.5 ± 0.58	5	4.67 ± 0.58	4.32 ± 0.69
Relevance	4 ± 1.73	4	4.57 ± 0.53	4 ± 0.82	4.25 ± 0.5	5	5	4.36 ± 0.76
Completeness	3	3	3.43 ± 0.98	3	3.5 ± 0.58	5	3.67 ± 0.58	3.36 ± 0.7
Sourcing	2.67 ± 0.58	3	2.71 ± 0.76	1	1.75 ± 0.96	1	3	2.28 ± 0.94
Usefulness	3.33 ± 1.15	3.67 ± 0.58	3.71 ± 0.76	2.75 ± 0.5	4	5	4 ± 1	3.64 ± 0.81
QAMAI total score (/30)	21.67± 4.93	22 ± 1	22.86 ± 3.34	19 ± 0.82	22.5 ± 2.38	26	25 ± 2	22.32 ± 3.08
	**Neurologist** [2]							
**QAMAI items** **(5-Likert Scale)**	**Indications** **(n = 3)**	**Surgical Procedure** **(n = 3)**	**Postoperative Care** **(n = 7)**	**Prognosis** **(n = 4)**	**Outcomes** **(n = 4)**	**Ethical Issues** **(n = 1)**	**Rehabilitation** **(n = 3)**	**Total** **(n = 25)**
Accuracy	4.67 ± 0.58	4.67 ± 0.58	4.29 ± 0.95	3.75 ± 0.5	4.25 ± 0.5	5	4.33 ± 0.58	4.32 ± 0.69
Clarity	4.67 ± 0.58	5	4.43 ± 0.53	4	4.75 ± 0.5	5	5	4.6 ± 0.5
Relevance	5	4.67 ± 0.58	3.71 ± 0.95	4	5	5	4.67 ± 0.58	4.6 ± 0.5
Completeness	4 ± 1	4 ± 1	4.67 ± 0.58	3.25 ± 0.5	3.25 ± 0.5	5	4	3.72 ± 0.79
Sourcing	3	3.67 ± 1.15	2.71 ± 0.49	2.75 ± 0.5	2.75 ± 0.5	3	3	2.92 ± 0.57
Usefulness	5	4.67 ± 0.58	4.43 ± 0.53	4	4.75 ± 0.5	5	4.33 ± 0.58	4.52 ± 0.51
QAMAI total score (/30)	26.33 ± 2.08	26.67 ± 3.51	24 ± 3.56	21.75 ± 0.96	24.75 ± 1.5	28	25.33 ± 0.58	24.68 ± 2.81
	**Neurosurgeon** [5]							
**QAMAI items** **(5-Likert Scale)**	**Indications** **(n = 3)**	**Surgical Procedure** **(n = 3)**	**Postoperative Care** **(n = 7)**	**Prognosis** **(n = 4)**	**Outcomes** **(n = 4)**	**Ethical Issues** **(n = 1)**	**Rehabilitation** **(n = 3)**	**Total** **(n = 25)**
Accuracy	4.33 ± 1.15	4.33 ± 0.58	4.14 ± 0.69	4.5 ± 0.58	4.25 ± 0.5	5	4.33 ± 0.58	4.32 ± 0.63
Clarity	5	4.33 ± 0.58	4.14 ± 0.69	3.5 ± 0.58	4.25 ± 0.5	5	4 ± 1	4.2 ± 0.71
Relevance	4.33 ± 0.58	4 ± 1	4.14 ± 0.69	4.25 ± 0.96	3.75 ± 0.96	5	4.33 ± 0.58	4.16 ± 0.8
Completeness	4	4.33 ± 0.58	3.71 ± 0.49	3.75 ± 0.5	3.75 ± 0.96	4	3.33 ± 0.58	3.8 ± 0.58
Sourcing	3.33 ± 0.58	4	3 ± 0.82	2.5 ± 0.58	2.75 ± 0.5	3	3.67 ± 0.58	3.12 ± 0.73
Usefulness	4 ± 1	3.33 ± 0.58	3.57 ± 0.79	3.75 ± 0.5	3.5 ± 0.58	5	4.33 ± 0.58	3.76 ± 0.72
QAMAI total score (/30)	25 ± 3	24.33 ± 2.52	22.71 ± 3.59	22.25 ± 1.71	22.25 ± 2.99	27	24 ± 2.65	23.36 ± 2.86
	**Neurosurgeon** [6]							
**QAMAI items** **(5-Likert Scale)**	**Indications** **(n = 3)**	**Surgical Procedure** **(n = 3)**	**Postoperative Care** **(n = 7)**	**Prognosis** **(n = 4)**	**Outcomes** **(n = 4)**	**Ethical Issues** **(n = 1)**	**Rehabilitation** **(n = 3)**	**Total** **(n = 25)**
Accuracy	4	3.33 ± 1.15	4.57 ± 0.53	4.5 ± 0.58	4.25 ± 0.5	5	4.67 ± 0.58	4.32 ± 0.69
Clarity	4.67 ± 0.58	4 ± 1.73	5	5	4.25 ± 0.5	5	5	4.72 ± 0.68
Relevance	5	4.67 ± 0.58	4.86 ± 0.38	5	4 ± 1.41	5	5	4.76 ± 0.66
Completeness	5	3.67 ± 1.53	5	5	4.25 ± 0.96	5	5	4.72 ± 0.74
Sourcing	3	3	3	3.5 ± 1	3	3	3	3.08 ± 0.4
Usefulness	4.67 ± 0.58	4.67 ± 0.58	4.86 ± 0.38	5	4 ± 1.41	5	5	4.72 ± 0.68
QAMAI total score (/30)	26.33 ± 0.58	23.33 ± 5.51	27.29 ± 1.11	28 ± 1.41	23.75 ± 4.03	28	27.67 ± 0.58	26.32 ± 2.91

Each item is assessed with a 5-point Likert scale (strongly disagree, disagree, neutral, agree, strongly agree). Abbreviations: QAMAI = Quality Analysis of Medical Artificial Intelligence Score.

**Table 3:** Stability of GPT-4o’s responses.

QAMAI Items (5-Likert Scale)	Pearson	*p*-Value
Accuracy	0.408	0.001
Clarity	0.509	0.001
Relevance	0.469	0.001
Completeness	0.437	0.001
Sourcing	0.282	0.002
Usefulness	0.610	0.001
QAMAI total score (/30)	0.616	0.001

Abbreviations: QAMAI = Quality Analysis of Medical Artificial Intelligence Score.

## Data Availability

The original contributions presented in this study are included in the article/Supplementary Material. Further inquiries can be directed to the corresponding author.

## Supplementary Materials

The following supporting information can be downloaded at: https://www.mdpi.com/article/10.3390/brainsci15040391/s1, Supplemental File S1, Supplemental Results: GPT-4o’s first-round answers to the 25 questions submitted. Supplemental File S2, Supplemental Results: Answers from GPT-4o to the second round for the 25 questions submitted, seven days later.

## Glossary

Artificial Intelligence (AI)	Computer systems designed to simulate human intelligence, often used in analyzing data, automating tasks, or assisting in medical education.
ChatGPT/GPT-4o	Generative pre-trained transformer, a type of large language model by OpenAI, used here to answer medical questions for relatives of critically ill patients.
Decompressive Hemicraniectomy (DHC)	A neurosurgical procedure where part of the skull is removed to relieve intracranial pressure, commonly used for severe brain swelling after a stroke.
Flesch Reading Ease (FRE)	A readability test measuring text complexity, with lower scores indicating harder-to-read text. FRE is used to assess if medical explanations are accessible to laypersons.
Flesch–Kincaid Grade Level (FKG)	A readability index indicating the grade level required to understand a text, used to evaluate the accessibility of medical information provided by AI.
Gunning Fog Index (GFI)	A readability test for English text that estimates the years of formal education needed to understand the text at first read.
Intraclass Correlation Coefficient (ICC)	A statistical measure used to evaluate the reliability of raters or measurements, here applied to assess consistency among evaluators scoring AI-generated medical information.
Large Language Model (LLM)	A type of AI model trained on vast amounts of text data to generate human-like responses. Examples include ChatGPT and GPT-4o.
Malignant Middle Cerebral Artery Infarction (MMCAI)	A severe type of ischemic stroke involving brain swelling that may require surgery, like DHC, due to increased intracranial pressure.
Quality Analysis of Medical Artificial Intelligence (QAMAI)	A tool for evaluating the quality of health information provided by AI, including factors like accuracy, clarity, and usefulness.
Retrieval-Augmented Generation (RAG)	A technique in AI that retrieves information from external sources to improve the accuracy of generated responses.
Statistical Package for the Social Sciences (SPSS)	A software suite used for statistical analysis, here employed to analyze the reliability and quality of AI responses.

## Abbreviations

DHC	Decompressive hemicraniectomy
FRE	Flesch Reading-Ease
FKG	Flesch-Kincaid Grade
GFI	Gunning Fog Index
GPT	Generative pre-trained transformer
ICC	Intraclass correlation coefficient
LLM	Large language model
mDISCERN	Modified DISCERN
MMCAI	Malignant middle cerebral artery infarction
QAMAI	Quality Analysis of Medical Artificial Intelligence
RAG	Retrieval-augmented generation
SPSS	Statistical Package for the Social Sciences
