# Supplementary material for: AI-Driven Information for Relatives of Patients with Malignant Middle Cerebral Artery Infarction: A Preliminary Validation Study Using GPT-4o
Source: Brain Sci. 2025 Apr 11;15(4):391. doi: 10.3390/brainsci15040391 (PMC12026103; doi:10.3390/brainsci15040391)
Supplement: Supplementary file 1 [file brainsci-15-00391-s001.zip › Supplemental_2_Answsers_GPT4o_Round2.pdf]

1. ***What is a decompressive hemicraniectomy and why is it necessary in the case of a malignant middle cerebral artery stroke ?***

A decompressive hemicraniectomy is a surgical procedure in which a portion of the skull is temporarily removed to relieve pressure on the brain. This procedure is typically performed in emergency situations where there is significant brain swelling (edema) that could otherwise lead to severe brain damage or death.

Indications for Decompressive Hemicraniectomy

In the context of a malignant middle cerebral artery (MCA) stroke, decompressive hemicraniectomy may be necessary. A malignant MCA stroke refers to a large and severe ischemic stroke that affects the territory of the middle cerebral artery. This type of stroke often leads to extensive brain tissue damage and subsequent swelling.

The swelling associated with a malignant MCA stroke can cause increased intracranial pressure (ICP). Because the skull is a rigid structure, there is limited space for the brain to expand. As the brain swells, pressure increases within the skull, leading to the potential for:

- Brain herniation: This occurs when parts of the brain are forced out of their normal position, either downward through the foramen magnum (the opening at the base of the skull) or laterally under the falx cerebri. Brain herniation can compress vital brain structures, leading to loss of consciousness, severe neurological impairment, or death.
- Reduced blood flow to the brain: Increased ICP can lead to a decrease in cerebral perfusion pressure, further reducing blood flow to the brain and exacerbating brain injury.

Purpose of the Procedure

The primary purpose of a decompressive hemicraniectomy in this context is to:

1. Prevent brain herniation: By removing a portion of the skull, the brain has more room to swell without being forced into abnormal positions, reducing the risk of herniation.
2. Reduce intracranial pressure: The removal of part of the skull allows the swollen brain to expand outward rather than inward, helping to lower intracranial pressure and restore better blood flow to the brain.
3. Improve survival and outcomes: In patients with malignant MCA strokes, without intervention, the mortality rate is high due to the complications of brain swelling. Decompressive hemicraniectomy has been shown to improve survival and, in some cases, lead to better functional outcomes, although many patients may still have significant neurological deficits.

Risks and Considerations

While decompressive hemicraniectomy can be life-saving, it is not without risks. These include:

- Infection: Since the skull is opened, there is a risk of infection.
- Further neurological damage: Although the procedure aims to prevent severe damage, the underlying stroke has usually already caused significant brain injury, and the surgery itself carries risks of complications.
- Delayed brain reconstruction: After the swelling subsides, a second surgery is typically required to replace the bone flap (cranioplasty), which also carries risks.

  In summary, a decompressive hemicraniectomy is a critical intervention for patients experiencing malignant MCA stroke, designed to alleviate life-threatening brain swelling and reduce intracranial pressure.

2. *Are there any alternative treatments to decompressive hemicraniectomy for malignant MCA stroke?*

Decompressive hemicraniectomy is a well-established treatment for malignant middle cerebral artery (MCA) stroke, particularly in cases where there is significant cerebral edema that could lead to increased intracranial pressure (ICP) and potential brain herniation. However, alternative treatments and adjunctive therapies exist, although they may be less definitive or are used in combination with or before hemicraniectomy.

<u>1. Medical Management:</u>

- Osmotherapy: The use of hyperosmolar agents like mannitol or hypertonic saline to reduce intracranial pressure by drawing fluid out of the brain tissue.
- Barbiturate-induced coma: This can be used to reduce brain metabolism and ICP but is generally a last resort due to the high risk of side effects.
- Steroids: Historically used to reduce cerebral edema, but they are not recommended for stroke-related edema due to a lack of evidence for effectiveness and potential harm.
- Aggressive blood pressure management: Maintaining optimal cerebral perfusion pressure while preventing further hemorrhage or ischemic injury.

<u>2. Endovascular and Surgical Alternatives:</u>

- Thrombectomy: If the stroke is identified early, mechanical thrombectomy to remove the clot in the MCA can reduce the severity of the stroke and potentially prevent the development of malignant edema.
- Cranial windowing: Instead of a full hemicraniectomy, a smaller cranial window can sometimes be used, though this is less common and generally not as effective in severe cases.

<u>3. Hypothermia:</u>

- Therapeutic Hypothermia: Lowering the body temperature can reduce metabolic demand and brain swelling, though its use in stroke patients is still under investigation and is not yet a standard of care.

<u>4. Stem Cell Therapy:</u>

- Emerging experimental therapies include the use of stem cells to potentially reduce brain injury and promote recovery, although this is not yet a mainstream or widely accepted alternative.

<u>5. Rehabilitation and Supportive Care:</u>

- Rehabilitation: Intensive rehabilitation can be crucial post-stroke, though it is more of a post-acute treatment rather than an alternative to hemicraniectomy.
- Palliative care: In cases where aggressive treatment is not pursued, supportive care and palliative measures may be considered to maintain patient comfort.

<u>6. Monitoring and Serial Imaging:</u>

- Close monitoring: In some cases, very close monitoring with frequent neurological exams and serial imaging may be employed to gauge the progression of edema and decide the optimal timing for any intervention, whether surgical or medical.

While these alternatives exist, the effectiveness of many depends on the timing of intervention, the patient's overall health, and the severity of the stroke. Decompressive hemicraniectomy remains the most definitive treatment for managing refractory increased ICP due to malignant MCA stroke. In practice, many of these alternatives are considered adjuncts to or part of a stepped approach that may ultimately include decompressive surgery if other measures fail to control the edema and pressure.

3. ***Can the malignant MCA stroke worsen if decompressive hemicraniectomy is delayed, or do we have time to think about it?***

In the case of a malignant middle cerebral artery (MCA) stroke, delaying decompressive hemicraniectomy can indeed worsen the patient's prognosis. Malignant MCA stroke refers to a large ischemic stroke that results in significant brain swelling (cerebral edema) and a high risk of herniation, leading to death or severe disability.

<u>Key Points to Consider:</u>

- Time-Sensitive Nature: The pathophysiology of malignant MCA stroke involves rapid swelling of brain tissue, which can increase intracranial pressure (ICP) and lead to brain herniation. This process can occur within the first 48 hours after the onset of the stroke. Early intervention with decompressive hemicraniectomy is crucial to relieve the pressure and prevent further damage.

- Outcomes with Early vs. Late Surgery: Studies have shown that decompressive hemicraniectomy performed within 48 hours of stroke onset significantly improves survival and functional outcomes compared to delayed surgery. The longer the delay, the higher the risk of irreversible brain damage and poor neurological outcomes.

- Clinical Deterioration: The condition of patients with malignant MCA stroke can deteriorate rapidly. Early signs of worsening include decreased level of consciousness, worsening neurological deficits, and signs of increasing ICP. Once these symptoms are evident, the window for effective intervention may be narrowing quickly.

<u>Conclusion:</u>
Given the rapid progression and the severe consequences of malignant MCA stroke, there is limited time to deliberate on the decision for decompressive hemicraniectomy. It is generally advised to proceed with the surgery as early as possible if the patient meets the criteria for this intervention. Timely decision-making is critical to improving outcomes and preventing life-threatening complications.

4. ***How long will a decompressive hemicraniectomy procedure for malignant MCA stroke take?***

A decompressive hemicraniectomy for a malignant middle cerebral artery (MCA) stroke typically takes about 1.5 to 3 hours to perform. The exact duration can vary depending on several factors, including:

The severity of the stroke and the patient's condition: More severe cases may require additional time to manage complications or ensure that all necessary interventions are completed.

The surgeon's experience and technique: A highly experienced surgical team might perform the procedure more efficiently, though they will still ensure that no steps are rushed.

Preoperative and postoperative care: While the surgical procedure itself might take 1.5 to 3 hours, the total time in the operating room may be longer due to anesthesia preparation, positioning the patient, and postoperative care such as ensuring the patient is stable before leaving the operating room.

It's important to note that this is an emergency procedure, often done to save the patient's life, so the timing can vary depending on the specific circumstances.

5. ***What are the possible risks and complications associated with decompressive hemicraniectomy for malignant MCA stroke?***

Decompressive hemicraniectomy is a life-saving surgical procedure performed in cases of malignant middle cerebral artery (MCA) stroke, where there is significant brain swelling that could lead to increased intracranial pressure (ICP) and potentially fatal herniation. While the procedure can significantly improve survival rates, it also comes with potential risks and complications. These can be broadly categorized into those associated with the surgery itself and those related to the long-term outcomes.

Surgical Risks and Complications

- **Infection:** Postoperative infection, including wound infections, meningitis, or brain abscess, can occur.
- **Bleeding:** There is a risk of intracranial hemorrhage either during or after the surgery.
- **Seizures:** The procedure may lead to seizures, both immediately postoperatively and in the long term.
- **Cerebrospinal Fluid (CSF) Leakage:** Leakage of CSF through the surgical site can occur, which might require additional interventions.
- **Subdural Hygroma:** Accumulation of CSF in the subdural space can happen, which might require monitoring or intervention.
- **Hydrocephalus:** There is a risk of developing hydrocephalus, where there is an abnormal buildup of CSF in the brain, potentially necessitating a shunt placement.
- **Wound Healing Issues:** Poor wound healing can occur, especially in patients with pre-existing conditions like diabetes or in those who are immunocompromised.

Neurological and Functional Outcomes

- **Hemiparesis or Hemiplegia:** Patients often experience significant motor deficits, particularly weakness or paralysis on the side opposite the stroke.
- **Aphasia or Other Cognitive Deficits:** Depending on the side of the brain affected, patients might experience language difficulties, memory problems, or other cognitive impairments.
- **Altered Consciousness:** Some patients may have a reduced level of consciousness or altered mental state postoperatively.
- **Depression and Anxiety:** The psychological impact of the surgery and the resultant neurological deficits can lead to depression, anxiety, or other psychiatric conditions.
- **Postoperative Cognitive Decline:** Some patients may experience a decline in cognitive functions after surgery, which can impact quality of life.

Long-term Complications

- **Sinking Skin Flap Syndrome (SSFS):** This is a condition where the skin over the craniectomy site becomes concave, potentially leading to neurological symptoms like headaches, dizziness, or cognitive disturbances.
- **Paradoxical Herniation:** In cases where the bone flap is not replaced in a timely manner, atmospheric pressure changes can cause brain tissue to shift, leading to paradoxical herniation.
- **Bone Flap Resorption:** After the bone flap is reimplanted (in a procedure called cranioplasty), there is a risk of bone flap resorption, particularly in younger patients or those with infection.
- **Cosmetic Deformity:** The absence of a bone flap can lead to noticeable skull deformities, which can be distressing for the patient.

Quality of Life Considerations

- **Dependence on Care:** Many patients require long-term care due to the neurological deficits resulting from the stroke and the surgery, affecting their independence.
- **Reduced Life Expectancy:** While the surgery improves survival, some studies suggest that patients who undergo decompressive hemicraniectomy might have a reduced life expectancy due to the severity of the initial stroke and associated complications.

Psychosocial and Emotional Impact

- **Adjustment Disorders:** The sudden change in functional abilities can lead to significant emotional and psychological stress for both patients and their families.
- **Caregiver Burden:** The need for extensive care can place a significant burden on caregivers, impacting their mental and physical health.

Overall, while decompressive hemicraniectomy can be life-saving and may lead to better functional outcomes in some patients, it is associated with significant risks and potential complications. These should be carefully considered by the medical team and discussed with the patient and family before proceeding with the surgery.

6. ***How is the brain protected without the skull after decompressive hemicraniectomy?***

After a decompressive hemicraniectomy, where a portion of the skull is removed to relieve pressure on the brain, the brain is protected in several ways despite the absence of the skull:

- **Dura Mater:**

The dura mater, the tough outer membrane surrounding the brain, remains intact or is repaired after surgery. This provides a protective barrier between the brain and the outside environment, helping to shield it from infections and other external threats.

- **Scalp and Skin:**

The skin and soft tissue overlying the brain are sutured back into place, covering the exposed area. This creates a protective layer, preventing direct exposure of the brain to the

outside world. The scalp offers some mechanical protection and also serves as a barrier to bacteria and other pathogens.

- <u>**Monitoring and Care:**</u>

Patients who undergo hemicraniectomy are usually closely monitored in a hospital setting, often in an intensive care unit (ICU). This careful monitoring helps prevent complications like infection, brain swelling, or other issues that could arise from the brain's exposure.

- <u>**Helmet Protection:**</u>

Once the patient is stable and begins rehabilitation, a specially designed protective helmet may be used when the patient is mobile. This helmet is custom-fitted and provides additional protection to the area of the head where the skull is absent.

- <u>**Planned Cranioplasty:**</u>

In many cases, after the initial swelling or pressure in the brain has subsided, a second surgery called a cranioplasty is performed. During this procedure, the removed section of the skull (or a synthetic substitute) is replaced to restore the protective bony layer around the brain.

- <u>**Positioning and Activity Restrictions:**</u>

During the recovery period, patients are often positioned carefully in bed to minimize the risk of trauma to the unprotected area of the brain. Activity is also typically restricted to prevent falls or other impacts that could harm the exposed brain tissue.
These measures work together to protect the brain and support the patient's recovery following a decompressive hemicraniectomy.

7. ***After decompressive hemicraniectomy for malignant MCA stroke, when will relatives be allowed to see the patient?***

After a decompressive hemicraniectomy for malignant MCA (middle cerebral artery) stroke, the timing of when relatives can see the patient depends on several factors, including the hospital's policies, the patient's condition, and the time needed for post-operative care.

<u>Generally, after the surgery:</u>

- Immediate Post-Operative Period: The patient will typically be transferred to an intensive care unit (ICU) or a neuro-intensive care unit (neuro-ICU) for close monitoring. During this time, the patient will be closely observed for any signs of complications, such as swelling, bleeding, or changes in neurological status.

- Initial Stabilization: Once the patient is stable, the healthcare team may allow brief visits from close relatives. This can range from a few hours after surgery to the next day, depending on the patient's stability and the hospital's specific policies.

- Visitation Guidelines: Most hospitals have specific visitation guidelines, particularly in ICUs, which may limit the number of visitors and the duration of visits. It is common for hospitals to allow immediate family members to visit once the patient is stable and settled in the ICU.

- Communication with Healthcare Team: The surgical team or ICU staff will usually communicate with the family after the procedure, providing updates on the patient's condition and guidance on when visits might be appropriate.

It’s important for relatives to follow the hospital’s instructions and guidelines to ensure the patient's safety and recovery. The healthcare team will prioritize the patient’s immediate post-operative care and stability before allowing visitors.

8. ***After decompressive hemicraniectomy for malignant MCA stroke, how soon will the patient wake up?***

The timing of when a patient may wake up after a decompressive hemicraniectomy for a malignant middle cerebral artery (MCA) stroke can vary widely and depends on several factors. These factors include the extent of the brain injury, the timing of the surgery, the patient’s overall health, and the severity of the stroke.

General Considerations:

- Severity of the Stroke: A malignant MCA stroke is severe, and the damage to the brain can be extensive. This can delay recovery of consciousness.

- Pre-Surgical Condition: If the patient was already in a deep coma or had significant neurological deficits before the surgery, recovery of consciousness may take longer.

- Surgical Outcome: The success of the surgery in relieving brain swelling and preventing further damage influences recovery. If the surgery effectively reduces intracranial pressure, the patient may recover consciousness sooner.

- Postoperative Care: Intensive care and management post-surgery, including controlling brain swelling, preventing infections, and maintaining adequate blood flow to the brain, are crucial for recovery.

Expected Timeline:

- Immediate Post-Surgery: It is common for patients to remain in a medically induced coma immediately after surgery to control brain swelling and give the brain time to recover. During this time, the patient may not wake up.

- Days to Weeks: In some cases, patients may start to regain consciousness within a few days to weeks after surgery as the brain swelling decreases and the brain begins to recover. However, this can vary significantly.

- Longer Recovery: Some patients may take weeks to months to regain consciousness, particularly if the stroke caused significant brain damage or if there were complications.

Prognostic Factors:

- Neurological Status: Patients with less severe initial neurological deficits generally have a better chance of waking up sooner.

- Imaging Findings: MRI or CT scans showing the extent of brain damage can help predict recovery.

- Age and Comorbidities: Older patients and those with other health issues may have a longer recovery period.

Conclusion:

There is no fixed timeline for when a patient will wake up after a decompressive hemicraniectomy for a malignant MCA stroke. The recovery of consciousness can range from a few days to several weeks or even months, depending on individual factors. Continuous neurological assessments and imaging studies are important to monitor progress and adjust care plans accordingly.

9. ***During the coma period after malignant MCA stroke, can the patient hear me and how should I talk to them?***

During a coma, especially following a severe event like a malignant middle cerebral artery (MCA) stroke, a patient is typically unresponsive to external stimuli. However, there is ongoing debate and research on whether patients in a coma can hear or process sounds, including speech.

Can a Comatose Patient Hear?

- **Awareness in a Coma:** Some studies suggest that patients in a coma may have some level of awareness and may be able to hear sounds, including voices. However, their ability to understand or process these sounds is likely limited and varies depending on the severity of the brain injury.
- **Individual Variation:** The extent to which a comatose patient can hear or respond varies. Some patients may retain a degree of auditory processing, while others might not. It’s difficult to predict based on clinical observations alone.

How Should You Talk to a Comatose Patient?

1. **Speak Softly and Clearly:** Even though the patient may not respond, it’s good practice to speak softly and clearly. The tone should be gentle and comforting.
2. **Use Simple and Positive Language:** Talk about positive things, familiar subjects, or things you know might bring them comfort. Avoid complex or distressing topics.
3. **Describe What You’re Doing:** If you’re providing care or just being there, describe your actions to the patient. For example, "I'm holding your hand" or "I'm sitting beside you."
4. **Be Patient and Consistent:** Keep your speech consistent, using the same phrases or words of comfort regularly. This can create a sense of routine and familiarity.
5. **Include Family or Personal Stories:** Share memories, tell them about the day, or talk about things that are meaningful to them. This can provide a sense of connection.
6. **Use Non-Verbal Communication:** Holding their hand, gentle touch, or other forms of non-verbal communication can be comforting even if the patient does not consciously perceive it.

Emotional Support for You

It’s important to care for yourself during this time. Being present for a loved one in a coma can be emotionally taxing, and seeking support from family, friends, or professionals is crucial.

While we can't be certain if the patient hears or processes what is being said, many find comfort in knowing that they are providing emotional support during a critical time.

10. ***How long will the patient need to stay in the ICU and hospital after decompressive hemicraniectomy for malignant MCA stroke?***

The length of stay in the ICU and hospital after a decompressive hemicraniectomy for a malignant middle cerebral artery (MCA) stroke can vary widely depending on several factors, including the patient's overall health, the severity of the stroke, the presence of complications, and the speed of recovery.

ICU Stay:

- **Typical Duration:** Most patients will need to stay in the ICU for **7 to 14 days** after the surgery. This period is crucial for close monitoring of neurological status, intracranial pressure, and other vital functions.
- **Monitoring Focus:** During the ICU stay, the medical team will manage and monitor intracranial pressure, ventilation, blood pressure, and other critical parameters. The ICU stay might be prolonged if there are complications such as infections, respiratory issues, or secondary brain injury.

Total Hospital Stay:

- **Typical Duration:** The overall hospital stay can range from **2 to 4 weeks** but may be longer depending on the patient's recovery trajectory. Some patients might need longer stays if they require intensive rehabilitation or if there are complications.
- **Rehabilitation Needs:** Following the ICU stay, patients often move to a step-down unit or a specialized stroke unit where they continue to receive care. The focus shifts toward rehabilitation, including physical therapy, speech therapy, and occupational therapy, which can influence the length of the hospital stay.

Key Factors Affecting Duration:

- **Complications:** The presence of complications, such as infection, pneumonia, or seizures, can prolong both ICU and hospital stays.
- **Neurological Recovery:** The degree of neurological recovery and the patient's ability to participate in rehabilitation also significantly impact the duration of the hospital stay.
- **Age and Pre-existing Conditions:** Older patients or those with significant pre-existing health conditions might experience longer recovery times.

In summary, while ICU stays typically last about 1 to 2 weeks, the overall hospital stay can extend to 2 to 4 weeks or more, depending on the individual patient's recovery and complications.

11. *What type of care and support will be needed at home after decompressive hemicraniectomy for malignant MCA stroke?*

After a decompressive hemicraniectomy for malignant middle cerebral artery (MCA) stroke, the patient will require extensive care and support at home. The specifics depend on the severity of the stroke, the extent of neurological deficits, and the overall recovery progress. Here's a general overview of the care and support that might be needed:

1. Physical Support and Mobility Assistance:

- **Mobility Aids:** The patient may need assistance with walking and transfers (e.g., from bed to chair). This could include the use of wheelchairs, walkers, or canes.
- **Positioning:** Regular repositioning may be necessary to prevent pressure sores, especially if the patient has limited mobility or is bedridden.
- **Fall Prevention:** The home should be adapted to reduce fall risk, including removing tripping hazards and installing grab bars in key areas like bathrooms.

2. Rehabilitation Therapy:

- **Physical Therapy:** To help regain strength, balance, and coordination. Physical therapists will also help in teaching exercises to improve mobility and prevent complications like contractures.
- **Occupational Therapy:** To help the patient relearn daily activities (e.g., dressing, bathing) and adapt to any physical limitations.
- **Speech and Language Therapy:** If the stroke affected speech, language, or swallowing, therapy might be needed to improve communication skills and address dysphagia.

3. Cognitive and Emotional Support:

- **Cognitive Rehabilitation:** This may be needed to address issues with memory, attention, problem-solving, and other cognitive functions.
- **Psychological Support:** Patients may experience emotional changes, depression, or anxiety. Psychological counseling or psychiatric support may be necessary.
- **Family Education:** Family members will need education on how to support the patient emotionally, recognize signs of depression, and cope with the challenges of caregiving.

4. Medication Management:

- **Administering Medications:** The patient will likely be on a regimen of medications (e.g., anticoagulants, anticonvulsants, pain management). Caregivers need to manage these medications, ensuring they are taken correctly and on time.
- **Monitoring for Side Effects:** Watch for side effects of medications, such as increased risk of bleeding (for anticoagulants) or cognitive changes.

5. Nutritional Support:

- **Dietary Needs:** The patient may need a modified diet, especially if there are swallowing difficulties. A speech therapist or dietitian can guide the appropriate dietary modifications.
- **Feeding Assistance:** If the patient has severe swallowing issues, they may require feeding through a tube (e.g., nasogastric or PEG tube).

6. Wound Care:

- **Incision Care:** The surgical site requires monitoring and care to prevent infection. Caregivers should be trained on how to clean the wound and recognize signs of infection.
- **Helmet Use:** If part of the skull was removed and not yet replaced, the patient may need to wear a protective helmet to safeguard the exposed brain area.

7. Communication:

- **Assistive Devices:** If speech is severely affected, communication aids (e.g., communication boards, electronic devices) may be necessary.
- **Regular Communication:** Keeping open communication with healthcare providers, including neurologists, neurosurgeons, and rehabilitation specialists, is vital for ongoing management and adjustment of care plans.

8. Social Support:

- **Home Care Assistance:** Depending on the level of disability, the patient may require professional in-home care services, such as nursing care or a personal support worker.
- **Community Resources:** Engaging with support groups, both for the patient and caregivers, can provide emotional support and practical advice.

9. Monitoring for Complications:

- **Seizure Monitoring:** As patients are at risk for post-stroke seizures, caregivers need to be aware of seizure signs and how to respond.
- **Hydrocephalus:** Caregivers should also be trained to recognize symptoms of increased intracranial pressure or hydrocephalus, such as severe headaches, nausea, or confusion.

10. Legal and Financial Support:

- **Power of Attorney and Guardianship:** Depending on the cognitive status of the patient, legal considerations may need to be addressed, including setting up powers of attorney for healthcare and financial decisions.
- **Insurance and Benefits:** Ensuring the patient has access to the necessary resources, whether through insurance, disability benefits, or community services.

Care plans should be personalized based on the individual needs of the patient and adjusted as they recover and their condition changes. Collaboration with healthcare professionals is key to ensuring comprehensive care at home.

12. ***Will the patient need permanent assistance after decompressive hemicraniectomy for malignant MCA stroke?***

Whether a patient will need permanent assistance after a decompressive hemicraniectomy for a malignant middle cerebral artery (MCA) stroke depends on several factors, including the severity of the stroke, the extent of brain damage, the patient’s age, overall health, and the success of rehabilitation efforts.

Factors Influencing Long-Term Outcomes:

1. **Severity of Stroke**: A malignant MCA stroke is a severe form of ischemic stroke, often leading to significant brain damage. The extent of this damage is a major determinant of long-term outcomes.
2. **Extent of Brain Damage**: The MCA supplies blood to large portions of the brain. If the stroke damages critical areas related to motor function, speech, or cognition, the patient may have lasting impairments.
3. **Age and Pre-existing Health Conditions**: Younger patients with fewer health issues may recover better than older patients with comorbidities.
4. **Rehabilitation**: The intensity and quality of post-surgical rehabilitation, including physical, occupational, and speech therapy, are crucial. Some patients regain

significant function with extensive rehabilitation, while others may continue to have profound disabilities.

Potential Long-Term Outcomes:

- **Partial Recovery**: Some patients may regain enough function to perform daily activities independently or with minimal assistance. They might still have some deficits, such as weakness on one side of the body (hemiparesis), difficulty with speech (aphasia), or cognitive impairments, but these may be manageable with therapy and assistive devices.
- **Significant Disability**: Many patients, especially those with extensive brain damage, may have lasting disabilities. This could include severe motor deficits (hemiplegia), inability to speak or understand language, significant cognitive impairments, or issues with swallowing. These patients often require long-term care and assistance with daily activities.
- **Permanent Assistance**: In cases of severe disability, patients may need permanent assistance, ranging from help with daily tasks to full-time care. This could be provided by family members, in-home caregivers, or at a long-term care facility.

Conclusion:

Many patients who undergo decompressive hemicraniectomy for malignant MCA stroke will require some degree of long-term assistance. The extent of assistance needed varies widely based on individual recovery, but it is common for patients to need help with daily activities, and in severe cases, permanent care might be necessary.

13. ***When will the removed part of the skull be replaced after decompressive hemicraniectomy for malignant MCA stroke?***

After a decompressive hemicraniectomy for malignant MCA (middle cerebral artery) stroke, the removed part of the skull is typically replaced in a procedure called a cranioplasty. The timing of the cranioplasty can vary depending on several factors, including the patient's condition, the presence of brain swelling, infection risks, and overall recovery.

Here is a general timeline:

1. **Early Cranioplasty (Within 3 Months):**
   - Some neurosurgeons prefer to replace the bone flap relatively early, often within 6 to 12 weeks after the initial surgery, especially if the patient has stabilized and there are no complications like infections or ongoing brain swelling. Early cranioplasty may help in reducing the risk of complications associated with leaving the brain exposed for an extended period.
2. **Delayed Cranioplasty (After 3 Months):**
   - In cases where brain swelling takes longer to subside, or if there are complications such as infections or the patient is not stable enough for another surgery, the cranioplasty may be delayed. This can extend to several months after the initial surgery. The decision to delay is usually made to minimize the risk of complications and ensure the patient is in the best possible condition for the procedure.
3. **Customized Timing:**
   - The timing can be highly individualized based on the patient’s neurological recovery, overall health, and specific clinical circumstances. The treating neurosurgeon will make the decision in collaboration with the broader medical team.

In summary, the cranioplasty typically occurs between 6 weeks and 6 months after the initial decompressive hemicraniectomy, with the exact timing being influenced by the patient's recovery and absence of complications.

14. ***What are the chances of survival after decompressive hemicraniectomy for malignant MCA stroke?***

Decompressive hemicraniectomy (DHC) is a life-saving surgical procedure used in the management of malignant middle cerebral artery (MCA) stroke, which is characterized by severe brain swelling that can lead to increased intracranial pressure and potential brain herniation. This surgery involves removing a portion of the skull to allow the swollen brain tissue to expand without causing further damage.

Survival Rates

Survival rates after DHC for malignant MCA stroke are generally favorable, but they come with varying degrees of neurological outcomes. Here's what the research shows:

1. **Overall Survival**: The procedure significantly increases the chances of survival. Without surgery, mortality rates for patients with malignant MCA stroke can be as high as 80% due to uncontrolled brain swelling. With DHC, survival rates improve to around 50-70%.
2. **Age Considerations**: Age plays a significant role in outcomes. Younger patients (under 60 years) tend to have better survival and functional outcomes compared to older patients (over 60 years). For younger patients, survival rates can be as high as 80-90%, while for older patients, the survival rate tends to be lower, around 50-60%.

Functional Outcomes

While DHC improves survival, it is important to consider the neurological and functional outcomes post-surgery:

1. **Functional Dependency**: Many survivors of malignant MCA stroke who undergo DHC may have significant disabilities. A large proportion of patients may have moderate to severe functional dependency, as measured by the modified Rankin Scale (mRS).
2. **Quality of Life**: Some studies report that 40-50% of survivors may have a favorable outcome (defined as mRS ≤ 3, where 0-2 indicates slight to no disability and 3 indicates moderate disability). However, a substantial number of patients may have an mRS of 4-5, indicating severe disability but survival.
3. **Influencing Factors**: Factors such as the timing of surgery, the extent of brain infarction, pre-stroke functional status, and post-operative care significantly influence both survival and functional outcomes.

Conclusion

The chances of survival after decompressive hemicraniectomy for malignant MCA stroke are significantly improved compared to conservative management. However, the procedure is associated with varying degrees of neurological outcomes, with many survivors facing moderate to severe disabilities. The decision to proceed with DHC should be made considering the patient's age, baseline functional status, and the potential for meaningful recovery.

15. ***What is the functional prognosis after decompressive hemicraniectomy for malignant MCA stroke?***

The functional prognosis after decompressive hemicraniectomy for malignant middle cerebral artery (MCA) stroke can vary based on several factors, including the timing of surgery, patient age, extent of brain injury, and pre-existing medical conditions. Here’s a summary of what is generally known:

1. Survival Rates:

- **Increased Survival**: Decompressive hemicraniectomy has been shown to significantly reduce mortality rates in patients with malignant MCA stroke. Without surgery, mortality rates can approach 80%, whereas with surgery, survival rates improve to around 50-70%.

2. Functional Outcomes:

- **Moderate to Severe Disability**: Although decompressive hemicraniectomy improves survival, many survivors are left with moderate to severe disability. The modified Rankin Scale (mRS) is often used to measure functional outcomes, where a score of 0 indicates no symptoms, and 6 indicates death.
- **Range of Outcomes**:
    - **mRS 0-3 (Mild to Moderate Disability)**: Some patients achieve functional independence (mRS 0-3), although this is less common.
    - **mRS 4-5 (Severe Disability)**: A significant number of survivors have severe disability (mRS 4-5), requiring assistance with daily activities. This includes hemiparesis, aphasia, and cognitive impairments.
- **mRS 6 (Death)**: Despite surgery, some patients may not survive, though this is less common with timely intervention.

3. Factors Influencing Prognosis:

- **Age**: Younger patients (typically those under 60 years) tend to have better functional outcomes compared to older patients.
- **Timing of Surgery**: Early intervention (within 48 hours of stroke onset) is associated with better outcomes.
- **Extent of Stroke**: The larger the area of infarction and brain swelling, the worse the prognosis.
- **Pre-existing Conditions**: Comorbidities such as diabetes, hypertension, and previous strokes can negatively impact recovery.

4. Quality of Life Considerations:

- **Postoperative Rehabilitation**: Intensive rehabilitation plays a crucial role in maximizing functional recovery. However, the quality of life may still be significantly impacted.
- **Patient and Family Perspectives**: It's essential to consider the patient's and family’s views on acceptable outcomes when deciding on decompressive surgery, as some may prefer not to survive with a severe disability.

5. Long-term Outlook:

- **Variable Recovery**: Long-term recovery is variable, with some patients improving over time, while others may have persistent deficits.

- **Ongoing Care Needs**: Many patients will require long-term care and support, whether from family members or professional caregivers.

In summary, while decompressive hemicraniectomy can save lives after malignant MCA stroke, it often results in moderate to severe disability. The decision to proceed with surgery should be individualized, taking into account the potential for meaningful recovery, patient and family preferences, and the anticipated quality of life post-surgery.

16. What are the chances of a full recovery after decompressive hemicraniectomy for malignant MCA stroke?

Decompressive hemicraniectomy is a surgical procedure performed to relieve intracranial pressure by removing part of the skull. It is often used as a life-saving measure in patients with malignant middle cerebral artery (MCA) stroke, where brain swelling can be severe and lead to fatal outcomes.

Outcomes and Recovery:

The chances of a full recovery after decompressive hemicraniectomy for malignant MCA stroke vary widely and depend on several factors, including the patient's age, the extent of the stroke, the time to surgery, and overall health before the stroke.

1. **Mortality Rate**: Decompressive hemicraniectomy significantly reduces mortality rates in patients with malignant MCA stroke. Studies have shown that without surgery, mortality can be as high as 80%, but with surgery, it can drop to around 30-40%.
2. **Functional Outcome**:
    - **Moderate to Severe Disability**: While decompressive hemicraniectomy can be life-saving, it often results in survivors experiencing moderate to severe disability. Many patients will have long-term neurological impairments, including hemiplegia (paralysis on one side of the body), aphasia (language difficulties), and cognitive deficits.
    - **Good Recovery**: A full recovery, defined as regaining near-normal function and independence, is relatively rare. Younger patients (typically under 60 years old) have better chances of a favorable outcome, and early surgery within 48 hours of stroke onset is associated with better results.
    - **Poor Outcome**: Patients who are older, have a more extensive stroke, or present later for surgery are at higher risk of poor functional outcomes, including severe disability requiring long-term care.
3. **Long-Term Prognosis**:
    - The long-term prognosis varies. Some patients can regain a significant degree of independence with intensive rehabilitation, but many will require some level of assistance with daily activities.
    - Cognitive rehabilitation and physical therapy are critical components of recovery, and outcomes can improve over time, but the process is typically slow and can take months to years.
4. **Quality of Life**:
    - Quality of life after surgery is often compromised due to physical and cognitive limitations, though some patients report satisfactory quality of life despite these challenges. The perceived quality of life can differ between patients and caregivers, with patients often reporting a higher quality of life than caregivers might expect.

Conclusion:

While decompressive hemicraniectomy can save lives in the context of malignant MCA stroke, the chances of a full recovery are limited. Most survivors will have significant neurological impairments, though some may achieve a functional and relatively independent life with extensive rehabilitation, particularly if they are younger and the surgery is performed early. However, complete recovery to pre-stroke function is uncommon.

17. What factors influence the patient's recovery after decompressive hemicraniectomy for malignant MCA stroke?

Recovery after a decompressive hemicraniectomy (DHC) for malignant middle cerebral artery (MCA) stroke is influenced by a variety of factors. These factors can be broadly categorized into preoperative, intraoperative, and postoperative considerations. Below are the key factors that impact recovery:

1. Preoperative Factors

- **Patient Age:** Younger patients generally have better outcomes due to greater brain plasticity and a higher potential for neurological recovery.
- **Stroke Severity:** The extent of initial neurological deficits, as measured by scales like the National Institutes of Health Stroke Scale (NIHSS), can predict recovery potential. More severe strokes generally result in worse outcomes.
- **Time to Surgery:** Earlier intervention, typically within 48 hours of stroke onset, is associated with better outcomes as it helps reduce the extent of secondary brain injury due to swelling.
- **Comorbidities:** The presence of other medical conditions, such as diabetes, hypertension, or heart disease, can complicate recovery. Patients with fewer comorbidities tend to recover better.
- **Preoperative Neurological Status:** The patient's neurological status before surgery, including level of consciousness and motor function, can be predictive of outcomes. Patients with better preoperative status typically have better recovery.

2. Intraoperative Factors

- **Surgical Technique:** The extent of the craniectomy, ensuring sufficient bone removal to allow brain expansion, is crucial. Adequate decompression can minimize brain damage from swelling.
- **Intraoperative Complications:** The occurrence of complications such as excessive bleeding, infection, or unintended injury to the brain during surgery can negatively impact recovery.

3. Postoperative Factors

- **Postoperative Complications:** Complications like infection, seizures, hydrocephalus, or hemorrhage can adversely affect recovery. Effective management of these complications is vital.
- **Rehabilitation:** Early and intensive rehabilitation, including physical, occupational, and speech therapy, is critical in maximizing functional recovery. The availability and quality of rehabilitation services significantly influence outcomes.
- **Postoperative Monitoring and Care:** Close monitoring for signs of increased intracranial pressure, electrolyte imbalances, and other postoperative issues is essential. Proper ICU care can mitigate the risk of further neurological deterioration.
- **Family and Social Support:** A strong support system can greatly aid in the recovery process by ensuring adherence to rehabilitation and medical follow-ups and providing psychological support.

4. Neurological and Functional Outcomes

- **Extent of Initial Brain Damage:** The degree of damage to critical areas such as the motor cortex, language centers, and other functional areas influences long-term recovery.
- **Neuroplasticity:** The brain's ability to reorganize and form new connections, especially in younger patients, is a critical determinant of recovery.

5. Long-term Care and Rehabilitation

- **Ongoing Rehabilitation:** Long-term rehabilitation tailored to the patient's specific deficits (e.g., motor, speech, cognitive) is essential for maximizing recovery.
- **Use of Assistive Devices:** The availability and use of assistive technologies, like braces, wheelchairs, or communication devices, can improve quality of life and functional independence.
- **Psychosocial Support:** Addressing the psychological impact of the stroke, such as depression or anxiety, and ensuring social support are crucial for long-term recovery and quality of life.

6. Genetic and Biological Factors

- **Genetic Predispositions:** Some patients may have genetic factors that influence recovery, such as variations in genes related to neuroplasticity or response to injury.
- **Biological Factors:** Inflammatory responses, neurochemical changes, and hormonal factors also play a role in recovery, although these are less well understood.

In summary, recovery after a decompressive hemicraniectomy for malignant MCA stroke is influenced by a combination of the patient's preoperative status, the technical success of the surgery, the management of postoperative complications, and the quality and intensity of rehabilitation. Each of these factors can significantly impact the overall outcome and functional recovery of the patient.

18. ***How long will it take to see the maximum improvements in the patient's condition after decompressive hemicraniectomy for malignant MCA stroke?***

The timeline for seeing maximum improvements in a patient's condition after a decompressive hemicraniectomy for a malignant middle cerebral artery (MCA) stroke varies depending on several factors, including the severity of the stroke, the patient's overall health, and the timing of the surgery. However, there are general trends observed in the recovery process:

1. **Immediate Postoperative Period (First Few Days to Weeks):**
   - **Initial Stabilization:** In the first few days to weeks, the primary focus is on stabilizing the patient and managing postoperative complications such as infection, hemorrhage, or cerebral edema.
   - **Early Signs of Improvement:** Some patients may show early signs of neurological improvement, such as better consciousness levels or reduced need for ventilatory support, but these are often modest.
2. **Early Recovery Phase (Weeks to 3 Months):**
   - **Gradual Neurological Improvement:** Patients typically experience gradual improvements in neurological function during the first few months post-surgery. This period is critical for rehabilitation efforts, including physical, occupational, and speech therapy.

   - **Assessing Long-Term Potential:** By 3 months, healthcare providers can often better assess the patient's long-term recovery potential, although further improvements are still possible.
3. **Late Recovery Phase (3 Months to 1 Year or More):**
   - **Continued Recovery:** Significant neurological improvements can continue up to 6 to 12 months after surgery. Recovery tends to plateau after the first year, although slow, incremental gains can still occur.
   - **Maximal Functional Recovery:** Most patients reach their maximum functional recovery within the first year, though some may continue to improve in specific areas beyond this time frame.

Key Points:

- **Maximum Improvements:** While significant improvements are typically observed within the first 6 to 12 months, the exact timeline can vary widely among patients.
- **Rehabilitation:** Active participation in rehabilitation programs is crucial for optimizing recovery and achieving the best possible outcomes.

The prognosis and extent of recovery will depend on factors such as the patient's age, pre-existing health conditions, the severity of the initial stroke, and the effectiveness of rehabilitation efforts. Regular follow-up with healthcare providers is essential to monitor progress and adjust treatment as necessary.

19. What is the long-term impact on the patient's cognitive abilities after decompressive hemicraniectomy for malignant MCA stroke?

Decompressive hemicraniectomy is a surgical procedure used to relieve intracranial pressure in patients suffering from a malignant middle cerebral artery (MCA) stroke, which is a severe form of ischemic stroke that can lead to extensive brain swelling and potentially fatal outcomes. While this procedure can be lifesaving and can significantly reduce mortality, the long-term impact on a patient's cognitive abilities is a critical consideration in the overall assessment of outcomes.

Long-Term Cognitive Impact

1. **Survival with Disability**:
   - Patients who undergo decompressive hemicraniectomy for malignant MCA stroke often survive, but many are left with significant disabilities, including cognitive impairments. The extent of cognitive deficits can vary widely depending on the severity of the stroke, the timing of the surgery, and the area of the brain affected.
2. **Global Cognitive Decline**:
   - Many survivors experience a decline in global cognitive functions, including memory, attention, executive functions, and processing speed. These impairments can be severe, especially if the dominant hemisphere is affected, which can impact language and verbal memory in addition to other cognitive domains.
3. **Specific Cognitive Domains**:
   - **Memory and Attention**: Patients may have significant problems with both short-term and long-term memory, as well as difficulties in maintaining attention and concentration.
   - **Executive Functions**: Planning, problem-solving, and multitasking abilities are often impaired, which can affect daily living and the ability to return to work.

- **Language and Communication**: When the dominant hemisphere is affected, patients may experience aphasia, a condition that affects the ability to communicate effectively.

4. **Quality of Life**:
   - Cognitive impairments contribute significantly to reduced quality of life. Many patients may require long-term care or rehabilitation and may struggle with activities of daily living, leading to dependence on caregivers.
5. **Individual Variability**:
   - There is considerable variability in outcomes, with some patients regaining a significant degree of cognitive function, while others may suffer from profound cognitive deficits. This variability is influenced by factors such as age, overall health, the extent of brain damage, and the timing of the intervention.
6. **Rehabilitation and Recovery**:
   - Intensive neurorehabilitation can help improve cognitive functions over time, but recovery is often partial. The brain's plasticity allows for some compensation, but many deficits may persist.

Conclusion

While decompressive hemicraniectomy can be a lifesaving procedure for patients with malignant MCA stroke, the long-term cognitive outcomes are often characterized by significant impairments. These deficits can impact multiple cognitive domains, leading to a reduced quality of life and increased dependence on care. The decision to proceed with this intervention should involve careful consideration of the potential for cognitive and functional outcomes, along with discussions with the patient's family about the long-term implications. Early and continuous rehabilitation efforts are essential to maximize cognitive recovery and improve overall outcomes.

20. Will the patient be able to recognize his relatives after decompressive hemicraniectomy for malignant MCA stroke?

Decompressive hemicraniectomy is a surgical procedure performed to relieve intracranial pressure by removing part of the skull. It is often done in cases of malignant middle cerebral artery (MCA) stroke to prevent brain herniation and death. Whether a patient will be able to recognize relatives after such a procedure depends on several factors:

1. **Extent of Brain Injury:** The outcome heavily depends on the extent of the brain damage caused by the stroke before the surgery. Malignant MCA strokes often lead to extensive damage in the areas of the brain responsible for various functions, including language, cognition, and recognition of faces.
2. **Location of the Stroke:** If the stroke affects the right hemisphere of the brain, which is typically involved in facial recognition, the patient may have difficulty recognizing people, even close relatives. If the left hemisphere is affected, the patient may have more difficulties with language and communication.
3. **Recovery and Rehabilitation:** The ability to recognize relatives may improve over time with rehabilitation. Neuroplasticity, or the brain's ability to reorganize and form new connections, can sometimes compensate for lost functions to a degree.
4. **Post-Surgical Complications:** Complications such as infections, seizures, or further brain swelling can impact cognitive recovery and recognition abilities.
5. **Individual Differences:** The patient's overall health, age, and pre-existing cognitive reserve play a significant role in recovery. Younger patients and those with fewer pre-existing conditions may recover better.

In summary, while some patients may retain or regain the ability to recognize relatives after a decompressive hemicraniectomy for a malignant MCA stroke, the extent and speed of recovery are highly variable and depend on multiple factors. It is best assessed on a case-by-case basis in consultation with the treating neurologist and rehabilitation specialists.

21. ***Are there any aids to daily living that will be needed after decompressive hemicraniectomy for malignant MCA stroke?***

After a decompressive hemicraniectomy for malignant middle cerebral artery (MCA) stroke, a patient may require various aids to assist with daily living due to potential impairments resulting from the stroke and surgery. These aids and supports will depend on the specific deficits the patient experiences, such as motor, sensory, cognitive, or speech difficulties. Here are some common aids and supports that might be needed:

1. Mobility Aids:

- **Wheelchair:** If there is significant hemiparesis or hemiplegia (weakness or paralysis on one side of the body), a wheelchair might be necessary.
- **Walker or Cane:** For those who retain some mobility but need support to maintain balance.
- **Leg Braces or Orthotics:** To help with foot drop or other mobility issues related to weakness or spasticity.

2. Hand and Arm Aids:

- **Splints or Braces:** To support weak limbs, prevent contractures, or assist with hand function.
- **Adaptive Utensils:** Specially designed forks, spoons, and knives with larger grips or angled handles to help with eating.
- **Reachers or Grabbers:** To help pick up objects without bending or overreaching.

3. Communication Aids:

- **Speech Generating Devices:** If there is aphasia or significant speech impairment, electronic communication devices may be useful.
- **Communication Boards:** Simple boards with pictures or letters that the patient can point to.

4. Cognitive Aids:

- **Memory Aids:** Devices like calendars, alarms, or apps to help with memory deficits.
- **Organizational Tools:** Planners or to-do lists to help with executive function challenges.

5. Activities of Daily Living (ADL) Aids:

- **Dressing Aids:** Devices like button hooks or zipper pulls can assist with dressing.
- **Bathing and Toileting Aids:** Grab bars, shower chairs, raised toilet seats, and commodes can assist with bathroom safety and independence.
- **Adaptive Clothing:** Clothing with Velcro fasteners instead of buttons or zippers.

6. Environmental Modifications:

- **Home Modifications:** Widened doorways, ramps, stairlifts, and accessible bathroom modifications may be necessary depending on mobility and independence levels.
- **Bed and Seating Adjustments:** Adjustable beds, pressure-relieving cushions, and special mattresses may help with positioning and comfort.

7. Therapeutic Support:

- **Physical Therapy:** To aid in recovery of motor functions and improve mobility.
- **Occupational Therapy:** To help with regaining independence in daily activities.
- **Speech Therapy:** For assistance with communication, swallowing, and cognitive functions.

8. Psychological and Social Support:

- **Counseling Services:** To help with the emotional and psychological impact of the stroke and surgery.
- **Social Services:** To assist with accessing resources, financial support, and caregiving needs.

The specific aids and supports needed will depend on the individual's recovery progress and the extent of the stroke's effects. A multidisciplinary team, including neurologists, physical therapists, occupational therapists, and speech therapists, typically assesses the patient's needs and recommends appropriate aids.

22. ***What are the ethical considerations for withdrawing life support if necessary after decompressive hemicraniectomy for malignant MCA stroke?***

Withdrawing life support after a decompressive hemicraniectomy for malignant middle cerebral artery (MCA) stroke involves complex ethical considerations. These decisions often intersect with medical, legal, cultural, and personal values, and it's essential to approach them with sensitivity and care. Here are some key ethical considerations:

1. Autonomy and Informed Consent

- **Respecting Patient Autonomy:** The patient's previously expressed wishes (e.g., through an advance directive or conversations) should be central to any decision. If the patient is unable to express their wishes, the surrogate decision-maker (often a family member) should be guided by what the patient would have wanted.
- **Informed Decision-Making:** Ensuring that the surrogate decision-makers are fully informed about the prognosis, potential outcomes, and implications of continuing or withdrawing life support is crucial. They should understand the likelihood of meaningful recovery, as well as the risks of continued treatment.

2. Best Interests of the Patient

- **Quality of Life Considerations:** The decision should consider the likely quality of life if life support is continued. For instance, if continued life support would lead to a state the patient would have found unacceptable (e.g., severe disability, loss of consciousness), it may be ethically justifiable to withdraw support.
- **Suffering and Burden:** The potential for ongoing suffering and the burden of treatment on the patient should be weighed. Prolonging life in a state of suffering without the prospect of recovery can be ethically problematic.

3. Proportionality and Futility

- **Proportionality:** The benefits of continued life support should be proportionate to the burdens it imposes. If the burdens (e.g., pain, distress, or a vegetative state) outweigh the benefits (e.g., survival with a meaningful quality of life), withdrawal may be ethically appropriate.
- **Medical Futility:** If the medical team determines that continued life support would be futile (i.e., it would not achieve the desired outcome of recovery or meaningful life), it may be ethical to consider withdrawing it.

4. Non-Maleficence and Beneficence

- **Non-Maleficence (Do No Harm):** The principle of non-maleficence suggests that life support should not be continued if it only serves to prolong suffering or a poor quality of life.
- **Beneficence (Acting in the Patient's Best Interest):** Actions should aim to promote the well-being of the patient. If life support is not contributing to the patient's well-being, withdrawing it may align with this principle.

5. Legal and Institutional Guidelines

- **Legal Considerations:** Decisions about withdrawing life support must comply with relevant legal standards, which can vary by jurisdiction. In some places, there may be specific laws or court precedents governing the withdrawal of life support.
- **Institutional Policies:** Hospitals and medical institutions often have policies and ethics committees to guide these decisions. Consulting with an ethics committee can provide additional support and clarity in complex cases.

6. Cultural and Religious Considerations

- **Cultural Sensitivity:** Different cultures have varying beliefs about end-of-life care. Understanding and respecting these cultural perspectives is important in the decision-making process.
- **Religious Beliefs:** The patient's and family's religious beliefs may strongly influence their views on life support and its withdrawal. Engaging with spiritual advisors or chaplains can help navigate these beliefs.

7. Communication and Compassion

- **Transparent Communication:** Clear, compassionate, and honest communication with the patient's family or surrogates is essential. They should be given time to understand the situation, ask questions, and express their concerns.
- **Support for Families:** The process of withdrawing life support is emotionally challenging for families. Providing psychological and emotional support during this time is ethically important.

8. Continuity of Care

- **Palliative Care:** If life support is withdrawn, ensuring that the patient receives appropriate palliative care to manage symptoms and maintain comfort is critical. The focus should shift to providing dignity in dying.

In summary, the decision to withdraw life support after a decompressive hemicraniectomy for malignant MCA stroke involves balancing respect for the patient's autonomy, the principles

of beneficence and non-maleficence, the proportionality of treatment, and cultural, religious, and legal considerations. It requires a multidisciplinary approach, involving physicians, nurses, ethicists, and possibly legal counsel, to ensure that the decision is made in the best interests of the patient.

23. What does rehabilitation consist of after decompressive hemicraniectomy for malignant MCA stroke and how long will it take?

Rehabilitation after a decompressive hemicraniectomy for a malignant middle cerebral artery (MCA) stroke is a critical aspect of recovery, focusing on maximizing the patient's functional abilities and improving their quality of life. The rehabilitation process is typically multidisciplinary, involving physical therapy, occupational therapy, speech therapy, and neuropsychological support. The length and intensity of rehabilitation depend on the extent of the stroke, the patient's pre-stroke health, and their response to therapy.

Components of Rehabilitation

1. **Physical Therapy (PT):**
    - **Mobility Training:** This involves exercises to help the patient regain strength and coordination in their limbs, especially on the side of the body affected by the stroke.
    - **Balance and Coordination:** Exercises to improve balance, reduce the risk of falls, and enhance coordination.
    - **Gait Training:** For patients who have difficulty walking, gait training with assistive devices (e.g., walkers, canes) may be necessary.
    - **Strengthening Exercises:** To improve overall muscle strength, particularly in the affected limbs.
2. **Occupational Therapy (OT):**
    - **Daily Living Skills:** OT focuses on helping the patient regain the ability to perform activities of daily living (ADLs), such as dressing, bathing, eating, and grooming.
    - **Adaptive Strategies:** Teaching patients how to use adaptive equipment and strategies to cope with physical limitations.
    - **Fine Motor Skills:** Exercises to improve dexterity and hand-eye coordination, which are often impaired after a stroke.
3. **Speech and Language Therapy (SLT):**
    - **Speech Rehabilitation:** For patients with aphasia (difficulty speaking or understanding language), speech therapy can help improve communication skills.
    - **Swallowing Therapy:** Dysphagia (difficulty swallowing) is common after a stroke, and speech therapists help patients regain safe swallowing function.
    - **Cognitive-Communication Therapy:** To help with memory, attention, and problem-solving skills.
4. **Neuropsychological Support:**
    - **Cognitive Rehabilitation:** To address memory, attention, and executive function deficits.
    - **Emotional and Psychological Support:** Depression, anxiety, and emotional lability are common after a stroke, and psychological support is crucial for mental health.
5. **Nutritional Support:**
    - Many patients require tailored dietary plans to meet their nutritional needs, especially if they have swallowing difficulties.
6. **Social and Vocational Support:**

  - Reintegration into the community and, when possible, returning to work are long-term goals. Social workers and vocational therapists may assist with these aspects.

Duration of Rehabilitation

- **Acute Phase (0-3 months):** The most intensive rehabilitation usually occurs in the first three months following the stroke. During this time, the brain is most adaptable, and significant gains can be made.
- **Subacute Phase (3-6 months):** Rehabilitation continues, though the intensity might decrease. Patients often transition from inpatient to outpatient therapy.
- **Chronic Phase (6 months and beyond):** Rehabilitation may continue on an outpatient basis, focusing on maintaining and improving the gains made. Some patients might continue to require therapy for years, particularly if they have significant deficits.

The duration of rehabilitation varies widely. Some patients may show substantial recovery within 6 to 12 months, while others may require ongoing therapy and support for several years. In some cases, full recovery to pre-stroke functionality may not be possible, and rehabilitation will focus on maximizing independence and quality of life.

Prognosis and Recovery Time

Recovery after a malignant MCA stroke can be prolonged and is highly variable. Some patients may regain significant function within a year, while others may experience permanent disabilities. The extent of rehabilitation needed typically correlates with the severity of the stroke and the patient's response to therapy. Continuous reassessment by the rehabilitation team will guide the ongoing care and adjustments to the rehabilitation plan.

24. ***How can family members support the patient's rehabilitation at home after decompressive hemicraniectomy for malignant MCA stroke?***

Supporting a patient's rehabilitation at home after a decompressive hemicraniectomy for malignant MCA (Middle Cerebral Artery) stroke requires a comprehensive approach that addresses the physical, emotional, and cognitive challenges they may face. Here's a guide for family members to help facilitate the recovery process:

1. Understanding the Patient's Condition:

- **Educate Yourself**: Learn about the patient's specific condition, the effects of the stroke, and the implications of the hemicraniectomy. Understanding the recovery trajectory and potential complications can help you provide better care.
- **Follow Medical Advice**: Adhere strictly to the care instructions provided by the medical team, including medication schedules, dietary recommendations, and physical therapy routines.

2. Creating a Safe and Comfortable Environment:

- **Modify the Home Environment**: Ensure the home is safe and accessible. This might involve installing grab bars, removing tripping hazards, and setting up a bedroom and bathroom on the ground floor if mobility is an issue.
- **Comfort and Rest**: Provide a quiet and comfortable space for the patient to rest. Proper rest is crucial for brain recovery.

3. Physical Rehabilitation:

- **Assist with Exercises**: Support the patient in following a prescribed physical therapy regimen. This may include helping with exercises to improve strength, balance, and coordination.
- **Mobility Assistance**: Help with transfers, walking, and other mobility tasks as recommended by healthcare professionals. Use assistive devices if necessary, such as walkers or wheelchairs.

4. Cognitive and Speech Therapy:

- **Engage in Cognitive Exercises**: Encourage activities that stimulate cognitive function, such as puzzles, reading, or memory games, under the guidance of a therapist.
- **Support Speech Therapy**: If speech is affected, practice exercises provided by a speech-language therapist. Simple communication, patience, and encouragement are key.

5. Emotional and Psychological Support:

- **Offer Emotional Support**: Be patient and compassionate. Recovery can be slow, and the patient may experience frustration or depression. Provide reassurance and positive reinforcement.
- **Professional Counseling**: Consider professional counseling or support groups for both the patient and family members to cope with emotional stress.

6. Encouraging Independence:

- **Promote Self-care**: Encourage the patient to perform self-care activities as independently as possible, such as dressing, grooming, and eating. This boosts confidence and fosters independence.
- **Set Realistic Goals**: Work with healthcare providers to set achievable rehabilitation goals. Celebrate small milestones to keep the patient motivated.

7. Nutritional Support:

- **Balanced Diet**: Ensure the patient follows a nutritious diet that supports healing and overall health. If swallowing is an issue, prepare meals that are easy to eat and swallow.
- **Hydration**: Keep the patient well-hydrated, monitoring fluid intake as necessary.

8. Medication Management:

- **Monitor Medications**: Keep track of all medications, ensuring they are taken as prescribed. Watch for any side effects and report them to the healthcare provider.

9. Regular Follow-up Care:

- **Attend Appointments**: Ensure the patient attends all follow-up appointments with neurologists, surgeons, therapists, and other healthcare professionals.
- **Monitor Progress**: Keep a log of the patient's progress, noting any changes in symptoms or abilities, and communicate these to the healthcare team.

10. Seek Support for Caregivers:

- **Caregiver Support**: Caring for a stroke patient can be physically and emotionally demanding. Seek support from other family members, friends, or professional caregivers to avoid burnout.
- **Respite Care**: Consider respite care options if needed, to ensure that primary caregivers have time to rest and recharge.

11. Plan for Long-Term Care Needs:

- **Long-Term Planning**: Discuss and plan for the patient's long-term needs, including the possibility of requiring ongoing therapy, home care, or adapting the living situation.

Supporting a loved one through rehabilitation after a decompressive hemicraniectomy for a malignant MCA stroke is a challenging but crucial task. With the right approach, patience, and support, family members can play a vital role in the patient's recovery and improvement in quality of life.

25. Are there any new or emerging rehabilitation techniques that could benefit the patient after decompressive hemicraniectomy for malignant MCA stroke?

Yes, there are several emerging rehabilitation techniques and approaches that could benefit patients recovering from decompressive hemicraniectomy following a malignant middle cerebral artery (MCA) stroke. Some of these techniques are still in the experimental stages or early phases of clinical research, but they show promise in improving outcomes:

1. Neurostimulation Techniques

- **Transcranial Magnetic Stimulation (TMS):** TMS is a non-invasive method that uses magnetic fields to stimulate nerve cells in the brain. It has shown potential in enhancing motor recovery by promoting plasticity in the brain regions adjacent to the infarcted area. Some studies suggest that TMS, when applied to the unaffected hemisphere, can reduce interhemispheric inhibition and facilitate recovery of the affected side.
- **Transcranial Direct Current Stimulation (tDCS):** This technique involves delivering a low electrical current to the brain, which can modulate neuronal activity. tDCS has been explored as a means to enhance motor and cognitive recovery post-stroke by improving cortical excitability and plasticity.

2. Robotic-Assisted Rehabilitation

- **Robotic Exoskeletons:** These devices support and guide limb movements during rehabilitation exercises, enabling repetitive and precise motor practice. Exoskeletons can help retrain motor pathways and improve upper and lower limb function. Some devices are combined with virtual reality (VR) to enhance engagement and feedback.
- **Robotic Gait Training:** Specifically for hemiplegic patients, robotic gait trainers can assist in re-establishing walking patterns and improving gait symmetry by providing controlled, repetitive, and intensive practice.

3. Virtual Reality (VR) and Augmented Reality (AR)

- **VR-Based Rehabilitation:** VR offers immersive environments where patients can engage in exercises tailored to their specific deficits. It has been shown to improve motor function, balance, and even cognitive skills by providing engaging and motivating environments that can adapt to the patient's progress.
- **AR-Based Rehabilitation:** AR can overlay digital information onto the real world, allowing patients to practice motor skills in a more integrated and interactive environment. This approach can help with motor, cognitive, and visuospatial rehabilitation.

4. Constraint-Induced Movement Therapy (CIMT)

- CIMT is an established technique, but newer adaptations and protocols are emerging. CIMT involves restraining the unaffected limb to encourage use of the

affected limb. Intensive practice with the affected limb can lead to significant improvements in motor function. Emerging protocols are exploring varying intensity and duration, combined with other rehabilitation modalities.

5. Mirror Therapy

- Mirror therapy involves using a mirror to create a visual illusion of movement in the affected limb by moving the unaffected limb. This technique can help in regaining motor function, particularly in the upper limbs, by stimulating the brain's motor areas and enhancing neuroplasticity.

6. Task-Oriented Training

- Task-oriented training focuses on practicing specific activities or tasks that are meaningful to the patient's daily life. This approach helps in improving functional outcomes by targeting the neural circuits involved in those specific tasks, thus promoting better generalization to real-life activities.

7. Stem Cell Therapy

- While still largely experimental, stem cell therapy is being investigated as a potential treatment to enhance recovery after stroke. The idea is that stem cells could help repair damaged brain tissue and improve outcomes when combined with traditional rehabilitation techniques.

8. Pharmacological Interventions

- **Neuroprotective Agents:** Some pharmacological agents are being studied for their potential to enhance neuroplasticity and recovery when used in conjunction with rehabilitation. These include drugs that modulate neurotransmitter systems or promote neuronal growth and repair.
- **Early Mobilization and Intensive Rehabilitation:** Recent studies suggest that starting rehabilitation early and at a high intensity may enhance recovery, although the exact timing and intensity levels are still under investigation.

9. Biofeedback and Neuromuscular Electrical Stimulation (NMES)

- **Biofeedback:** This technique involves using sensors to provide real-time feedback on physiological functions, such as muscle activity, to help patients learn to control movements and improve motor function.
- **NMES:** NMES uses electrical impulses to stimulate muscle contractions, which can help in preventing muscle atrophy and promoting motor recovery. It can be used as part of a broader rehabilitation strategy.

10. Tele-rehabilitation

- Tele-rehabilitation involves delivering rehabilitation services remotely using telecommunication technologies. This approach has gained traction, especially in the post-COVID era, and allows continuous, supervised rehabilitation at home, which can be particularly beneficial for long-term recovery.

These emerging techniques often work best when integrated with traditional rehabilitation methods. Ongoing research is needed to fully understand their efficacy and how they can be optimized for individual patients.
