# Supplementary material for: AI-Driven Information for Relatives of Patients with Malignant Middle Cerebral Artery Infarction: A Preliminary Validation Study Using GPT-4o
Source: Brain Sci. 2025 Apr 11;15(4):391. doi: 10.3390/brainsci15040391 (PMC12026103; doi:10.3390/brainsci15040391)
Supplement: Supplementary file 1 [file brainsci-15-00391-s001.zip › Supplemental_1_Answsers_GPT4o_Round1.pdf]

1. **What is a decompressive hemicraniectomy and why is it necessary in the case of a malignant middle cerebral artery stroke ?**

A decompressive hemicraniectomy is a surgical procedure that involves removing a portion of the skull to relieve pressure on the brain. This operation is typically performed in cases of severe brain swelling (edema) that cannot be controlled by other means. The procedure allows the swollen brain to expand outward rather than being compressed within the fixed volume of the skull, thereby reducing intracranial pressure and preventing further brain damage.

In the context of a malignant middle cerebral artery (MCA) stroke, this procedure becomes necessary due to the following reasons:

Malignant Middle Cerebral Artery Stroke

1. Severe Brain Swelling: A malignant MCA stroke refers to a large and severe type of ischemic stroke that occurs when the middle cerebral artery, one of the major arteries supplying blood to the brain, becomes blocked. This type of stroke often leads to significant brain swelling.

2. Increased Intracranial Pressure: The infarcted (damaged) brain tissue swells, increasing intracranial pressure (ICP). The rigid structure of the skull limits the space available, leading to compression of brain structures.

3. Herniation Risk: As the pressure continues to rise, the brain can herniate, which means brain tissue is forced through openings in the skull or across structures within the skull. This can lead to severe neurological damage and is often fatal.

Why Decompressive Hemicraniectomy is Necessary

1. Relieves Pressure: By removing a portion of the skull, decompressive hemicraniectomy provides space for the swollen brain to expand outward. This helps to reduce intracranial pressure and prevents the brain from being compressed within the skull.

2. Prevents Further Brain Damage: Lowering the intracranial pressure can prevent secondary damage to brain tissues that are still healthy. This helps in preserving as much neurological function as possible.

3. Improves Outcomes: Clinical studies have shown that in cases of malignant MCA stroke, early decompressive hemicraniectomy can significantly improve survival rates and outcomes, including reducing the risk of severe disability.

Procedure Overview

1. Surgical Approach: The surgeon makes an incision in the scalp and removes a portion of the skull, typically on the side where the stroke has occurred. The removed bone flap is usually stored for possible reattachment later.

2. Dura Opening: The dura mater, a protective membrane covering the brain, is also opened to allow the brain to expand.

3. Post-Surgical Care: After the surgery, patients require intensive care to monitor and manage intracranial pressure, prevent infections, and support overall neurological recovery.

4. Follow-Up Surgery: Once the brain swelling has subsided, a follow-up surgery called cranioplasty is often performed to replace the removed bone flap or to insert a synthetic material to protect the brain.

In summary, decompressive hemicraniectomy is a life-saving procedure in the context of a malignant MCA stroke, addressing the critical issue of increased intracranial pressure and preventing catastrophic outcomes due to brain herniation.

2. **Are there any alternative treatments to decompressive hemicraniectomy for malignant MCA stroke?**

Yes, there are several alternative treatments to decompressive hemicraniectomy for malignant middle cerebral artery (MCA) stroke, though the options and their effectiveness can vary based on the patient's specific condition. Here are some alternatives:

1. Medical Management:
   - Osmotherapy: Agents like mannitol or hypertonic saline can be used to reduce intracranial pressure.
   - Blood Pressure Management: Maintaining optimal blood pressure to ensure adequate cerebral perfusion.
   - Neuroprotective Agents: Experimental treatments aimed at protecting brain tissue from damage.

2. Endovascular Therapy:
   - Mechanical Thrombectomy: Removal of the clot causing the stroke through a catheter-based procedure. This is typically most effective when performed within a few hours of symptom onset.

3. Therapeutic Hypothermia:
   - Lowering the body temperature to reduce metabolic demand and limit secondary brain injury. This is still under investigation for stroke patients.

4. Corticosteroids:
   - Although controversial and not routinely recommended, corticosteroids have been used in some cases to reduce brain swelling.

5. Cerebral Spinal Fluid (CSF) Drainage:
   - Insertion of a ventricular catheter to drain CSF and reduce intracranial pressure.

6. Intravenous Thrombolysis:
   - Administration of tissue plasminogen activator (tPA) within a specific time window after stroke onset to dissolve the clot.

7. Anticoagulation and Antiplatelet Therapy:
- Use of medications to prevent further clot formation, though this is more of a preventative strategy than an acute treatment.

8. Barbiturate Coma:
- Inducing a coma using barbiturates to decrease cerebral metabolism and intracranial pressure.

The choice of treatment depends on various factors, including the timing of stroke onset, the patient's overall health, the extent of the stroke, and available resources. Decompressive hemicraniectomy is generally considered when there is significant brain swelling and medical management alone is insufficient to control intracranial pressure.

3. **Can the malignant MCA stroke worsen if decompressive hemicraniectomy is delayed, or do we have time to think about it?**

In cases of malignant middle cerebral artery (MCA) stroke, the timing of decompressive hemicraniectomy can be critical. Malignant MCA stroke is characterized by rapid and extensive brain swelling, which can lead to increased intracranial pressure (ICP), herniation, and death if not managed promptly.

Key points about the timing and effects of decompressive hemicraniectomy:

1. Early Intervention:
- Early decompressive hemicraniectomy, typically within 48 hours of stroke onset, is associated with better outcomes. Studies have shown that early surgery can significantly reduce mortality and improve functional outcomes.
- Delaying the procedure can result in worsening brain edema, increased risk of herniation, and potentially irreversible brain damage.

2. Critical Time Window:
- The decision to perform decompressive hemicraniectomy should ideally be made within the first 24 to 48 hours after stroke onset. This period is considered a critical time window for intervention.
- Beyond this window, the risks associated with the procedure may increase, and the potential benefits may decrease.

3. Assessment and Monitoring:
- Continuous monitoring of the patient's neurological status, imaging studies (e.g., CT or MRI scans), and ICP measurements can help guide the timing of surgery.
- If signs of increased ICP or deteriorating neurological status are observed, expedited surgical intervention is often necessary.

4. Consultation and Decision-Making:
- While it's important to involve the patient’s family in decision-making, the urgency of the situation often requires quick and decisive action by the medical team.

- Multidisciplinary consultation, including neurologists, neurosurgeons, and critical care specialists, is crucial to ensure the best possible outcome.

Conclusion:

Delaying decompressive hemicraniectomy in the context of malignant MCA stroke can lead to worsening outcomes. Therefore, while it is important to consider all factors and involve the patient's family in discussions, the decision to proceed with surgery should be made promptly within the critical time window to maximize the chances of a favorable outcome.

4. **How long will a decompressive hemicraniectomy procedure for malignant MCA stroke take?**

A decompressive hemicraniectomy for a malignant middle cerebral artery (MCA) stroke typically takes between 90 minutes to 3 hours. The duration can vary based on factors such as the patient's condition, the extent of the stroke, the complexity of the surgery, and the experience of the surgical team.

The procedure involves:

1. Preparation: Anesthesia and patient positioning, typically taking 30-45 minutes.
2. Craniotomy: Removing a portion of the skull to access the brain, which can take about 30-60 minutes.
3. Brain Decompression: Opening the dura (the outer membrane covering the brain) to relieve pressure, which can take around 30-60 minutes.
4. Closure: Reattaching the bone flap (sometimes not immediately replaced) and closing the surgical site, which can take 30-45 minutes.

These times are approximate and can vary significantly based on individual patient factors and intraoperative findings.

5. **What are the possible risks and complications associated with decompressive hemicraniectomy for malignant MCA stroke?**

Decompressive hemicraniectomy is a surgical procedure used to relieve intracranial pressure in patients experiencing malignant middle cerebral artery (MCA) stroke. While it can be life-saving and improve functional outcomes, it is associated with several risks and complications:

Early Postoperative Complications

1. Intracranial Hemorrhage :
   - Bleeding can occur at the surgical site or within the brain tissue, leading to increased intracranial pressure and potentially worsening neurological outcomes.

2. Infection :
   - Risk of infection at the surgical site (wound infection) or deeper (meningitis, brain abscess).

3. Hydrocephalus :
- Accumulation of cerebrospinal fluid (CSF) within the ventricles, requiring additional interventions like ventriculoperitoneal shunting.

4. Brain Herniation :
- Despite the decompression, shifting of brain structures can occur, leading to further brain damage.

5. Seizures :
- Increased risk of seizures postoperatively due to brain irritation or injury from the surgery.

6. Cerebral Edema :
- Worsening or persistent swelling of the brain can continue to pose a threat despite the decompressive surgery.

Long-Term Complications

1. Skull Defect (Cranial Deformity) :
- Absence of the bone flap leads to an asymmetrical skull, which can be cosmetically concerning and may require cranioplasty for reconstruction.

2. Neurological Deficits :
- Persistent or worsened neurological deficits, such as hemiparesis, aphasia, or cognitive impairments, depending on the extent and location of brain damage.

3. Subdural Hygromas :
- Collection of CSF in the subdural space, which can cause additional pressure on the brain and may require surgical drainage.

4. Postoperative Cognitive and Psychological Issues :
- Changes in behavior, cognition, and mood, which may significantly impact quality of life and require ongoing rehabilitation and support.

5. Bone Flap Complications :
- Infections or resorption of the replaced bone flap (if a cranioplasty is performed), leading to further surgeries.

Specific Considerations for Malignant MCA Stroke

- Massive Brain Swelling :
  - Malignant MCA stroke causes significant brain swelling, which can complicate the surgical procedure and postoperative recovery.

- Timing of Surgery :
  - The effectiveness of decompressive hemicraniectomy is highly dependent on the timing of the surgery. Delays can result in irreversible brain damage and poorer outcomes.

- Age and Comorbidities :
  - Older patients and those with significant comorbidities may have higher risks of complications and poorer outcomes.

General Surgical Risks

- Anesthesia-related complications :
  - Cardiovascular and respiratory complications associated with anesthesia.

- General surgical risks :
  - Risks of deep vein thrombosis (DVT), pulmonary embolism (PE), and other general postoperative complications.

Overall, decompressive hemicraniectomy can be a critical intervention for patients with malignant MCA stroke, but it carries significant risks and requires careful consideration of the potential benefits and complications. Multidisciplinary management and thorough preoperative assessment are crucial for optimizing outcomes.

6. **How is the brain protected without the skull after decompressive hemicraniectomy?**

After a decompressive hemicraniectomy, where a portion of the skull is removed to alleviate intracranial pressure, the brain is protected through several means:

1. Scalp Closure: The skin and soft tissue covering the skull are sutured back in place over the area where the bone flap was removed. This provides a protective barrier to the brain.

2. Duraplasty: The dura mater, which is the thick membrane that covers the brain, is often expanded or reinforced with a dural graft. This can help protect the brain from external forces and infections.

3. Temporary Synthetic Coverings: Sometimes, synthetic materials such as a titanium mesh or custom-made prosthetic materials are used temporarily to cover the exposed area. These materials provide additional protection and structural support.

4. Careful Monitoring: Patients undergo close neurological and medical monitoring in a hospital setting to ensure that any complications such as infection or increased intracranial pressure are promptly addressed.

5. Physical Protection Measures: Patients are advised to avoid activities that could lead to head trauma. Helmets or other protective headgear might be used during recovery to provide additional external protection when the patient is mobile.

6. Subsequent Surgery: After the brain swelling has subsided, a second surgery, called a cranioplasty, is typically performed to replace the bone flap or to insert a synthetic substitute to restore the integrity of the skull.

These measures collectively help to protect the brain during the period between the initial hemicraniectomy and the eventual cranioplasty.

7. **After decompressive hemicraniectomy for malignant MCA stroke, when will relatives be allowed to see the patient?**

After a decompressive hemicraniectomy for a malignant MCA (middle cerebral artery) stroke, the time when relatives will be allowed to see the patient can vary depending on several factors:

1. Immediate Post-Operative Period: Initially, the patient will be closely monitored in the recovery room or intensive care unit (ICU). During this critical period, which can last several hours, family visits might be restricted to ensure the patient stabilizes.

2. Hospital Policies: Different hospitals have varying policies regarding visiting hours and ICU access. Some hospitals allow family members to visit as soon as the patient is stable, while others may have more structured visiting times.

3. Patient's Condition: The severity of the stroke and the patient's condition post-surgery will influence when visits are allowed. If the patient is in a stable condition, family visits might be allowed sooner.

4. Communication with Medical Team: It's essential for relatives to communicate with the medical team for specific guidance. The healthcare providers can give the most accurate information regarding when visits are appropriate based on the patient's progress and hospital regulations.

Generally, family members can expect to be allowed to see the patient once the immediate post-operative period has passed and the patient is transferred to a stable environment, either in the ICU or a regular hospital room. This usually happens within the first 24 hours post-surgery, but the exact timing can vary.

8. **After decompressive hemicraniectomy for malignant MCA stroke, how soon will the patient wake up?**

The timing for a patient to wake up after a decompressive hemicraniectomy for a malignant middle cerebral artery (MCA) stroke can vary widely and depends on several factors:

1. Severity of the Stroke: The extent of brain damage due to the stroke itself is a significant determinant. More extensive damage can result in a longer recovery time.

2. Pre-existing Health Conditions: Patients with other health conditions may have a prolonged recovery period.

3. Complications During or After Surgery: Complications such as brain swelling, infections, or additional strokes can affect recovery time.

4. Age and Overall Health: Younger patients and those in better health generally recover faster.

5. Initial Neurological Status: Patients with better neurological function before surgery tend to wake up sooner.

In general, patients may start to show signs of waking up within a few days to weeks after surgery. Some patients might regain consciousness sooner, while others might take longer, and unfortunately, some may not wake up at all. It's crucial to have a multidisciplinary team, including neurologists, neurosurgeons, and critical care specialists, closely monitor and manage the patient during this critical period.

Recovery is a gradual process, and initial waking up might be followed by a period of confusion and disorientation. Rehabilitation and supportive care play a vital role in the patient's overall recovery.

Each patient's situation is unique, so it's essential to discuss the specific case with the treating medical team for more accurate prognostic information.

9. **During the coma period after malignant MCA stroke, can the patient hear me and how should I talk to them?**

During the coma period following a malignant MCA (middle cerebral artery) stroke, it is possible that the patient may have some level of awareness, although this can vary greatly depending on the severity and specifics of their condition. Research and anecdotal evidence suggest that some patients in a coma can hear and understand conversations happening around them, even if they cannot respond.

How to Talk to a Comatose Patient

1. Speak Normally and Respectfully: Talk to the patient as you normally would, with a calm and reassuring tone. Avoid speaking in a way that you wouldn't if they were conscious.

2. Identify Yourself: Start by introducing yourself every time you speak to them. This helps orient the patient and reinforces your presence.

3. Use Positive and Reassuring Language: Offer words of encouragement, support, and love. Let them know they are cared for and that you are there for them.

4. Talk About Familiar Topics: Discuss familiar subjects, such as family, friends, hobbies, or favorite memories. This can provide comfort and a sense of normalcy.

5. Read to Them: Reading aloud from a book, newspaper, or letters from friends and family can be comforting and engaging.

6. Play Their Favorite Music: Music can be a powerful tool in reaching someone in a coma. Play their favorite songs or calming music.

7. Touch: Gentle touch, like holding their hand or a light touch on their arm, can be soothing. Ensure this is done in a comforting manner.

8. Update Them: Keep them informed about what is happening in their surroundings and in the lives of their loved ones. It helps maintain a connection to the world outside their condition.

9. Be Patient: Understand that they may not respond, but your presence and voice can still have a positive impact.

10. Avoid Negative or Distressing Topics: Steer clear of subjects that might cause distress or anxiety.

Importance of Communication

Communicating with a comatose patient can have several benefits:
- Emotional Support: It provides emotional support to the patient and helps maintain a connection.
- Stimulation: Auditory and sensory stimulation can be beneficial for brain activity.
- Comfort: Familiar voices and sounds can provide comfort and reassurance.

It's essential to maintain hope and continue to talk to the patient, as hearing familiar and caring voices can be a significant source of comfort during their recovery process.

10. How long will the patient need to stay in the ICU and hospital after decompressive hemicraniectomy for malignant MCA stroke?

The length of stay in the ICU and the hospital after a decompressive hemicraniectomy for a malignant middle cerebral artery (MCA) stroke can vary significantly depending on several factors, including the patient's overall health, the severity of the stroke, the presence of complications, and the patient's recovery progress. However, general guidelines and averages can provide some insight:

ICU Stay
1. Initial Monitoring: Patients typically stay in the ICU for intensive monitoring and management immediately after surgery. This period is critical for monitoring neurological status, managing intracranial pressure, and addressing any postoperative complications.
2. Duration: The ICU stay can range from a few days to several weeks. On average, patients might spend about 1-2 weeks in the ICU, but this can be longer if complications arise or if the patient’s condition is particularly severe.

Hospital Stay
1. Post-ICU Care: After stabilization in the ICU, patients are usually transferred to a step-down unit or a general ward for continued recovery and rehabilitation.
2. Overall Hospital Stay: The total hospital stay, including the ICU stay, can vary widely. Many patients may need to stay in the hospital for 2-4 weeks, but some might require a longer stay if their recovery is slow or if they have additional medical issues that need to be addressed.

Rehabilitation
1. Inpatient Rehabilitation: After discharge from the acute care hospital, many patients will need to undergo inpatient rehabilitation, which can last several weeks to months depending on the extent of their disabilities and their recovery progress.
2. Outpatient Rehabilitation: Long-term recovery often involves outpatient rehabilitation services, including physical, occupational, and speech therapy.

Individual Variability
- Patient Factors: Age, baseline functional status, comorbidities, and the severity of the stroke can all influence the length of stay in both the ICU and the hospital.
- Complications: The development of complications such as infections, seizures, or additional strokes can extend the length of stay.
- Recovery Trajectory: The patient's neurological recovery trajectory plays a significant role in determining the length of stay and the need for ongoing rehabilitation.

It's important to note that each patient's recovery journey is unique, and these time frames are general estimates. Close coordination with the healthcare team, including neurosurgeons, neurologists, ICU specialists, and rehabilitation therapists, is essential to provide the best individualized care plan and to adjust expectations based on the patient's specific circumstances.

11. **What type of care and support will be needed at home after decompressive hemicraniectomy for malignant MCA stroke?**

After a decompressive hemicraniectomy for a malignant middle cerebral artery (MCA) stroke, comprehensive care and support are crucial for the patient's recovery. Here's an overview of the care and support that may be needed at home:

1. Medical Care
- Medication Management: Regular administration of prescribed medications such as anticoagulants, antiplatelets, blood pressure medications, and any other necessary drugs.
- Monitoring for Complications: Watch for signs of infection, increased intracranial pressure, seizures, and other potential complications.
- Wound Care: Proper care of the surgical site to prevent infection and ensure healing.
- Follow-up Appointments: Regular visits to the neurosurgeon, neurologist, and other specialists as recommended.

2. Rehabilitation
- Physical Therapy: To improve mobility, strength, balance, and coordination. This may include exercises, gait training, and other therapeutic activities.
- Occupational Therapy: To assist in regaining independence in daily activities such as dressing, bathing, cooking, and using adaptive equipment.
- Speech and Language Therapy: If the stroke has affected speech or swallowing, therapy to help regain these skills will be essential.
- Cognitive Therapy: To address any cognitive deficits such as memory, attention, problem-solving, and executive function.

3. Activities of Daily Living (ADLs) Support
- Personal Care Assistance: Help with bathing, dressing, grooming, and toileting as needed.
- Mobility Assistance: Ensuring safe movement around the home, preventing falls, and possibly using mobility aids like walkers or wheelchairs.
- Nutrition: Assistance with meal preparation and ensuring the patient receives a balanced diet tailored to their specific health needs. Monitoring for dysphagia (difficulty swallowing) if present.

4. Psychological and Emotional Support

- Counseling or Therapy: For both the patient and family to cope with the emotional impact of the stroke and surgery.
- Support Groups: Connecting with others who have experienced similar health issues can provide emotional support and practical advice.

5. Environmental Modifications
- Home Adaptations: Making necessary changes to the home environment to enhance safety and accessibility, such as installing grab bars, ramps, and modifying bathrooms.
- Assistive Devices: Using devices such as hospital beds, transfer benches, and adaptive utensils to aid daily living.

6. Education and Training for Caregivers
- Caregiver Training: Education on how to properly care for the patient, including recognizing signs of complications, performing physical therapy exercises, and using medical equipment.
- Emergency Preparedness: Knowing when and how to seek emergency medical help if needed.

7. Social Support
- Family and Friends: Encouraging involvement of family and friends for additional support and companionship.
- Community Resources: Utilizing local resources such as home health aides, respite care services, and transportation services.

8. Financial and Legal Assistance
- Insurance Navigation: Understanding and utilizing health insurance benefits for home health care and rehabilitation services.
- Legal Support: Addressing any legal matters such as power of attorney or advanced directives if not already in place.

A multidisciplinary approach involving healthcare professionals, caregivers, and family members will ensure the patient receives comprehensive care and support for optimal recovery and quality of life.

12. **Will the patient need permanent assistance after decompressive hemicraniectomy for malignant MCA stroke?**

Whether a patient will need permanent assistance after a decompressive hemicraniectomy for a malignant middle cerebral artery (MCA) stroke depends on several factors, including the extent of the stroke, the patient's age, pre-stroke health status, and the success of rehabilitation efforts. Here are some key points to consider:

1. Extent of the Stroke: Malignant MCA strokes often result in significant brain damage, affecting motor function, speech, and cognitive abilities. The more extensive the damage, the more likely the patient will need long-term assistance.

2. Age and Pre-Stroke Health: Younger patients and those in better health prior to the stroke tend to have better recovery outcomes. Older patients or those with pre-existing health conditions may have a more challenging recovery process.

3. Rehabilitation: The effectiveness and intensity of rehabilitation, including physical therapy, occupational therapy, and speech therapy, play crucial roles in determining the level of independence a patient can achieve.

4. Complications: Post-surgical complications, such as infections, seizures, or additional strokes, can affect the recovery process and the level of assistance needed.

5. Support System : A strong support system, including family, friends, and healthcare professionals, can significantly impact the patient's ability to regain independence.

In general, many patients who undergo decompressive hemicraniectomy for malignant MCA stroke do experience significant impairments and may require varying degrees of assistance, ranging from help with daily activities to full-time care. However, each patient's situation is unique, and ongoing evaluation and rehabilitation are essential to determining their long-term needs.

13. When will the removed part of the skull be replaced after decompressive hemicraniectomy for malignant MCA stroke?

After a decompressive hemicraniectomy for a malignant middle cerebral artery (MCA) stroke, the removed part of the skull is typically replaced in a procedure called cranioplasty. The timing of cranioplasty can vary depending on the patient's condition and recovery, but it generally occurs several weeks to a few months after the initial surgery. The decision on timing is influenced by factors such as:

1. Stability of the patient's neurological status: Ensuring the patient has stabilized and is not at risk of increased intracranial pressure or other complications.
2. Resolution of brain swelling: Waiting for the brain to return to a more normal size to avoid complications.
3. Absence of infection: Ensuring there are no ongoing infections or other medical issues that could complicate the procedure.
4. Overall health and recovery progress: Assessing the patient’s overall health status and readiness for another surgical procedure.

Typically, cranioplasty is performed around 6 to 12 weeks after the initial hemicraniectomy, but this can be adjusted based on the individual patient's circumstances and the treating medical team's judgment.

14. What are the chances of survival after decompressive hemicraniectomy for malignant MCA stroke?

The chances of survival after decompressive hemicraniectomy for malignant middle cerebral artery (MCA) stroke can vary based on several factors, including the patient's age, timing of the surgery, preexisting health conditions, and the extent of the stroke. However, some general trends have been observed in the medical literature:

1. Mortality Reduction: Decompressive hemicraniectomy significantly reduces mortality in patients with malignant MCA stroke. Studies indicate that the procedure can reduce mortality from approximately 70-80% without surgery to around 20-30% with surgery.

2. Functional Outcomes: While the surgery reduces mortality, the functional outcomes vary. Younger patients (under 60 years) tend to have better functional recovery compared to older patients.

3. Age Factor: Younger patients generally have a higher chance of survival and better functional outcomes. For patients over 60, the chances of survival are still improved, but they may have a higher likelihood of significant disability post-surgery.

4. Timing of Surgery: Early intervention (within 48 hours of stroke onset) is critical for better outcomes. Delays in surgery can lead to worse outcomes due to the progression of brain swelling and damage.

5. Preoperative Condition: Patients' condition before surgery, including their level of consciousness and presence of comorbidities, also plays a crucial role. Patients with better preoperative neurological status are more likely to have favorable outcomes.

Key Statistics from Studies

- DESTINY Trials (I and II): These trials showed that decompressive hemicraniectomy significantly reduces mortality and increases the number of patients with moderate disability or better outcomes.

- DECIMAL and HAMLET Trials: These also support the survival benefit of the surgery, although functional outcomes can vary widely.

Conclusion

Overall, decompressive hemicraniectomy for malignant MCA stroke can dramatically improve survival rates. However, it is important to weigh the benefits of survival against the potential for significant disability. Decisions should be individualized, taking into account the patient's age, pre-stroke functional status, and overall health.

For a specific prognosis, a detailed assessment by the treating medical team is essential, considering all individual factors and the latest evidence-based guidelines.

15. What is the functional prognosis after decompressive hemicraniectomy for malignant MCA stroke?

The functional prognosis after decompressive hemicraniectomy (DHC) for malignant middle cerebral artery (MCA) stroke varies depending on several factors, including the patient's age, the timing of the surgery, and the severity of the stroke. Here are some key points regarding the functional prognosis:

1. Overall Mortality and Morbidity Reduction: DHC significantly reduces mortality in patients with malignant MCA stroke. Studies have shown that without surgery, mortality rates can be as high as 80%, but with DHC, this can be reduced to around 20-30%.

2. Functional Outcome: While DHC improves survival, it does not guarantee good functional outcomes for all patients. Outcomes can range widely, with some patients achieving significant functional recovery and others remaining severely disabled.

3. Age-Related Outcomes: Younger patients tend to have better functional outcomes compared to older patients. Studies suggest that patients under the age of 60 have a higher likelihood of achieving functional independence post-surgery.

4. Timing of Surgery: Early intervention (within 48 hours of stroke onset) is associated with better outcomes. Delayed surgery is generally associated with poorer functional recovery.

5. Severity of Stroke and Preoperative Condition: The initial severity of the stroke and the patient's condition before surgery also play a significant role in the functional prognosis. Patients with less severe strokes and better preoperative conditions tend to have better outcomes.

6. Long-Term Functional Status: Long-term follow-up studies indicate that while some patients regain significant function, many survivors experience moderate to severe disability. Functional independence, defined by the ability to perform activities of daily living, is achieved in a subset of patients.

7. Quality of Life: Despite potential disabilities, some studies suggest that survivors can have an acceptable quality of life. The perception of quality of life can be subjective and varies among individuals and their caregivers.

8. Rehabilitation and Support: Intensive rehabilitation and support play a crucial role in the functional recovery of patients post-DHC. Multidisciplinary approaches involving physical therapy, occupational therapy, speech therapy, and psychological support are essential.

Summary of Prognostic Factors:

- Age: Younger patients have better outcomes.
- Timing: Early surgery (within 48 hours) improves prognosis.
- Stroke Severity: Less severe strokes have better outcomes.
- Preoperative Condition: Better initial condition correlates with improved recovery.
- Rehabilitation: Comprehensive rehabilitation is critical for functional recovery.

Prognosis in Numbers:

- Mortality Reduction: From 80% to 20-30% with DHC.
- Functional Independence: Approximately 20-40% of survivors achieve functional independence.
- Moderate to Severe Disability: A significant portion of survivors (up to 60-80%) may experience some level of disability.

Conclusion:
Decompressive hemicraniectomy for malignant MCA stroke significantly reduces mortality and can lead to various degrees of functional recovery. However, many patients may still experience moderate to severe disability, and the prognosis is better in younger patients, with early intervention and comprehensive post-surgical rehabilitation. The decision for DHC should be individualized, considering the patient's overall health, stroke severity, and potential for recovery.

16. What are the chances of a full recovery after decompressive hemicraniectomy for malignant MCA stroke?

The chances of a full recovery after a decompressive hemicraniectomy for a malignant middle cerebral artery (MCA) stroke vary significantly based on several factors, including the timing of the surgery, the patient's age, pre-stroke health, and the severity of the stroke. Here are some key points to consider:

Factors Influencing Recovery

1. Timing of Surgery: Early intervention within 48 hours of stroke onset has been shown to improve outcomes significantly.
2. Age: Younger patients tend to have better recovery outcomes compared to older patients.
3. Stroke Severity: The initial severity of the stroke and the extent of brain damage play critical roles in recovery.
4. Post-Operative Care: Comprehensive rehabilitation and supportive care post-surgery are crucial for improving functional outcomes.

General Outcomes

- Mortality Reduction: Decompressive hemicraniectomy significantly reduces mortality in patients with malignant MCA infarction. Studies have shown a reduction in mortality from around 70-80% to 30-40% with the procedure.
- Functional Outcomes: While the procedure improves survival rates, many survivors still experience significant disability. The degree of disability varies, with some patients achieving moderate independence and others requiring substantial assistance with daily activities.

Research Findings

Several studies have investigated the outcomes of decompressive hemicraniectomy for malignant MCA stroke:

1. DECIMAL, DESTINY, and HAMLET Trials: These trials demonstrated that decompressive hemicraniectomy reduces mortality and increases the number of patients achieving a moderate degree of functional independence.
2. Functional Independence: About 20-30% of survivors may achieve a level of independence where they can manage their own basic needs (modified Rankin Scale score of 3 or less).

Prognostic Factors

- Early Intervention: Performing the surgery within 48 hours of stroke onset is associated with better outcomes.
- Patient Age: Patients younger than 60 years tend to have better functional outcomes compared to older patients.
- Rehabilitation: Intensive and early rehabilitation can improve functional recovery and quality of life.

Conclusion

While decompressive hemicraniectomy can significantly reduce mortality in patients with malignant MCA stroke, achieving a full recovery is less common. Many patients experience varying degrees of disability post-surgery. Early intervention, younger age, and comprehensive post-operative rehabilitation are key factors that can improve the chances of a better functional outcome.

17. What factors influence the patient's recovery after decompressive hemicraniectomy for malignant MCA stroke?

Recovery after decompressive hemicraniectomy for malignant middle cerebral artery (MCA) stroke is influenced by several factors, including:

1. Patient Characteristics

- Age: Younger patients generally have better outcomes compared to older patients.
- Pre-stroke functional status: Patients with better functional status prior to the stroke tend to have better recovery.
- Comorbidities: The presence of other health conditions, such as diabetes, hypertension, or cardiovascular disease, can impact recovery negatively.

2. Stroke Characteristics

- Stroke Severity: The initial severity of the stroke, typically measured by the National Institutes of Health Stroke Scale (NIHSS), influences recovery. More severe strokes often result in poorer outcomes.

- Extent of Brain Damage: The volume of brain infarction and the involvement of critical brain areas (e.g., basal ganglia, internal capsule) play a crucial role. Larger infarctions usually correlate with worse prognosis.

3. Timing and Quality of Surgery
- Timing of Decompression: Earlier surgical intervention, ideally within 48 hours of stroke onset, is associated with better outcomes.
- Surgical Technique and Experience: The skill and experience of the surgical team, as well as the technique used, can affect the success of the surgery and recovery.

4. Postoperative Care
- Intensive Care: High-quality postoperative care, including intensive monitoring and management of intracranial pressure, is critical.
- Complication Management: Prompt identification and treatment of complications such as infections, seizures, or hydrocephalus are vital.

5. Rehabilitation
- Early and Intensive Rehabilitation: Initiating rehabilitation early and ensuring it is intensive and comprehensive can significantly improve functional outcomes.
- Access to Multidisciplinary Team: Access to a team of specialists, including physical therapists, occupational therapists, speech therapists, and neuropsychologists, enhances recovery.

6. Support Systems
- Family and Social Support: Strong support systems can provide emotional and practical assistance, which is important for recovery.
- Access to Resources: Availability of healthcare resources, rehabilitation facilities, and follow-up care can influence outcomes.

7. Neuroplasticity and Biological Factors
- Brain Plasticity: The brain's ability to reorganize and adapt following injury can impact recovery. Younger patients tend to have higher neuroplasticity.
- Genetic Factors: Genetic predispositions might influence the extent of recovery, though this area requires more research.

8. Psychosocial Factors
- Patient Motivation and Mental Health: A patient’s motivation, mental health status, and psychological resilience can affect their engagement in rehabilitation and overall recovery.

9. Treatment of Secondary Complications
- Management of Edema and Hemorrhage: Effective management of cerebral edema and prevention of hemorrhagic transformation is crucial.
- Vascular Health: Ensuring good overall vascular health to prevent recurrent strokes or vascular complications is important.

10. Follow-up and Long-term Care
- Regular Follow-up: Consistent follow-up with healthcare providers to monitor progress and adjust treatments as needed.
- Long-term Care Plans: Developing and adhering to long-term care plans tailored to the patient's needs, including lifestyle modifications, medication adherence, and ongoing therapy.

These factors interact in complex ways, and individual recovery can vary widely. Optimal management requires a holistic, patient-centered approach that addresses medical, functional, and psychosocial aspects of recovery.

18. How long will it take to see the maximum improvements in the patient's condition after decompressive hemicraniectomy for malignant MCA stroke?

The recovery timeline for a patient after decompressive hemicraniectomy for a malignant middle cerebral artery (MCA) stroke can vary widely based on individual factors such as the severity of the stroke, the patient's overall health, and how quickly they received treatment. However, general observations can be made:

1. Immediate Postoperative Period (First Few Days to Weeks) :
   - During the initial days and weeks following surgery, the focus is on stabilizing the patient, managing intracranial pressure, and preventing complications such as infections, seizures, and blood clots.
   - Some improvements in neurological function may be observed, but this period is primarily about recovery from the surgery itself and addressing any acute complications.

2. Subacute Period (Weeks to Months) :
   - Significant improvements often begin to manifest during this phase. Patients may start to regain some neurological functions as the brain begins to adapt to the changes and recover from the initial injury.
   - Intensive rehabilitation, including physical, occupational, and speech therapy, is crucial during this period to maximize recovery.

3. Long-term Recovery (Months to Years) :
   - The most substantial improvements are typically observed within the first 6 to 12 months post-surgery. However, recovery can continue for several years.
   - Neuroplasticity, the brain's ability to reorganize and form new neural connections, plays a significant role in long-term recovery. Patients can continue to make gains in function and quality of life with ongoing therapy and support.

4. Factors Influencing Recovery :
   - Age: Younger patients often have better recovery outcomes due to greater neuroplasticity.
   - Extent of the Stroke: The size and location of the stroke will impact the degree of recovery.
   - Preexisting Conditions: Patients with other health issues may have a slower recovery.
   - Rehabilitation: Access to and participation in intensive, multidisciplinary rehabilitation programs significantly affect the recovery trajectory.

In summary, while significant improvements can be seen in the first 6 to 12 months, recovery is an ongoing process that can extend over several years. Regular follow-ups and continuous rehabilitation are essential for maximizing the patient's potential for improvement.

19. What is the long-term impact on the patient's cognitive abilities after decompressive hemicraniectomy for malignant MCA stroke?

The long-term impact on cognitive abilities after decompressive hemicraniectomy (DHC) for malignant middle cerebral artery (MCA) stroke varies significantly among patients and is influenced by several factors, including the severity of the initial stroke, the timing of the surgery, and individual patient characteristics. Here are some key points regarding the potential cognitive outcomes:

1. Cognitive Improvement and Preservation :
   - Life-saving Intervention: DHC is primarily performed as a life-saving measure to relieve increased intracranial pressure and prevent brain herniation. Without this surgery, many patients would not survive, making any level of cognitive recovery a positive outcome in this context.

- Partial Cognitive Recovery: Some patients experience significant cognitive improvement and can regain various degrees of independence. This can include the recovery of language skills, executive functions, and memory, depending on the extent of brain injury and rehabilitation efforts.

2. Cognitive Deficits :
   - Common Deficits: Many patients may continue to experience long-term cognitive deficits after DHC. These can include impairments in attention, memory, executive function, language, and visuospatial abilities. The severity and type of cognitive deficits often correlate with the extent and location of the brain damage.
   - Quality of Life: Even with successful surgical outcomes, patients may face challenges in daily living activities and require ongoing support from caregivers and rehabilitation services.

3. Factors Influencing Cognitive Outcomes :
   - Age: Younger patients often have better neuroplasticity and may experience more significant cognitive recovery compared to older adults.
   - Severity of Stroke: The initial severity of the stroke and the extent of brain damage are critical factors. Larger infarcts and more severe strokes generally lead to worse cognitive outcomes.
   - Timing of Surgery: Early intervention with DHC can help minimize brain damage caused by increased intracranial pressure, potentially leading to better cognitive outcomes.
   - Rehabilitation: Intensive and prolonged rehabilitation, including physical, occupational, and speech therapy, can greatly influence the extent of cognitive recovery.

4. Studies and Research :
   - Heterogeneous Outcomes: Research indicates that cognitive outcomes after DHC for malignant MCA stroke are highly variable. Some studies report that a substantial proportion of survivors achieve functional independence, while others indicate persistent and significant cognitive and functional impairments.
   - Long-term Follow-up: Long-term follow-up studies are crucial to understanding the full extent of cognitive recovery or decline. Many patients show improvements in the first few months post-surgery, but long-term outcomes can vary, with some patients continuing to improve for years.

In summary, while decompressive hemicraniectomy can be life-saving for patients with malignant MCA stroke, the long-term impact on cognitive abilities varies widely. Patients may experience a range of outcomes, from significant cognitive recovery to persistent deficits, influenced by multiple factors including the initial severity of the stroke, age, and the extent of rehabilitation efforts.

20. Will the patient be able to recognize his relatives after decompressive hemicraniectomy for malignant MCA stroke?

The ability of a patient to recognize relatives after decompressive hemicraniectomy for malignant middle cerebral artery (MCA) stroke depends on several factors, including the extent of the brain damage caused by the stroke, the patient's pre-existing health condition, and the success of the surgery and subsequent rehabilitation.

Here are some key considerations:

1. Extent of Brain Damage: The MCA supplies a large portion of the brain, including areas involved in language, sensory perception, and motor control. Damage to these areas can

result in significant deficits. If the stroke affects the regions responsible for facial recognition or memory, it may impair the patient's ability to recognize relatives.

2. Surgical Outcome: Decompressive hemicraniectomy is performed to relieve intracranial pressure and prevent further brain damage. The success of this surgery in preventing additional damage can influence cognitive recovery.

3. Rehabilitation: Post-surgery rehabilitation plays a crucial role in recovery. Intensive therapy, including physical, occupational, and speech therapy, can help improve cognitive and functional abilities.

4. Individual Variability: Each patient's recovery is unique. Some patients may regain the ability to recognize relatives relatively quickly, while others may experience prolonged or permanent impairments.

5. Neuroplasticity: The brain's ability to reorganize itself and form new neural connections (neuroplasticity) can aid recovery. Younger patients generally have more neuroplasticity and may recover better than older patients.

In summary, while it is possible for a patient to regain the ability to recognize relatives after decompressive hemicraniectomy for a malignant MCA stroke, the outcome depends on multiple factors. Close monitoring, comprehensive rehabilitation, and supportive care are essential for optimizing recovery.

21. Are there any aids to daily living that will be needed after decompressive hemicraniectomy for malignant MCA stroke?

After a decompressive hemicraniectomy for a malignant MCA (middle cerebral artery) stroke, patients often require various aids and support for daily living. The specific aids needed depend on the severity of the stroke and the resulting deficits. Here are some common aids and supports that may be necessary:

1. Mobility Aids
- Wheelchairs: For those with significant mobility issues.
- Walkers or Rollators: For patients who can walk but need support.
- Canes: For those with minor balance issues.

2. Adaptive Equipment for Activities of Daily Living (ADLs)
- Grab Bars and Handrails: Installed in bathrooms and other areas for stability.
- Shower Chairs or Benches: For safety while bathing.
- Raised Toilet Seats: To assist with getting on and off the toilet.
- Long-handled Bath Sponges: To help with washing without bending.
- Reachers or Grabbers: To pick up objects without bending over.

3. Communication Aids
- Speech Generating Devices: For those with aphasia or severe speech impairments.
- Communication Boards or Apps: For non-verbal communication.

4. Cognitive Aids
- Memory Aids: Such as planners, alarms, or apps to assist with memory issues.
- Medication Management Systems: Pill organizers or automatic dispensers.

5. Feeding Aids
- Adaptive Utensils: Such as weighted or angled utensils for easier handling.
- Non-slip Mats: To prevent plates and bowls from sliding.

- Plate Guards or Divided Plates: To make eating easier and more manageable.

6. Home Modifications
- Ramps: For wheelchair access if there are steps.
- Widened Doorways: To accommodate wheelchairs or walkers.
- Lowered Counters and Shelves: To make items more accessible.

7. Assistive Technology
- Smart Home Devices: To control lights, temperature, and other features with voice commands.
- Automated Medication Dispensers: To help manage medications.

8. Personal Care Aids
- Dressing Aids: Such as button hooks, zipper pulls, and long-handled shoe horns.
- Specialty Clothing: Easy-to-wear clothes designed for those with limited mobility.

9. Therapeutic Equipment
- Range of Motion Devices: To prevent stiffness and improve mobility.
- Exercise Bands and Weights: For physical therapy exercises.

10. Support Services
- Home Health Care: Nurses, physical therapists, occupational therapists, and speech therapists.
- Caregiver Support: Assistance from trained caregivers for daily activities.

11. Cognitive Rehabilitation Tools
- Puzzles and Games: To stimulate cognitive function.
- Apps and Software: Designed to improve memory and cognitive skills.

12. Vision Aids
- Magnifiers: For those with visual impairments.
- Large Print Books and Materials: To assist with reading.

The specific needs of each patient will vary, and it is essential to conduct a comprehensive assessment with healthcare professionals such as occupational therapists, physical therapists, and speech therapists to determine the appropriate aids and modifications.

22. What are the ethical considerations for withdrawing life support if necessary after decompressive hemicraniectomy for malignant MCA stroke?

Withdrawing life support after decompressive hemicraniectomy for malignant middle cerebral artery (MCA) stroke involves several ethical considerations:

1. Autonomy
   - Patient's Wishes: Respecting the previously expressed wishes of the patient regarding end-of-life care is crucial. This includes living wills or advance directives that outline the patient's preferences for life-sustaining treatments.
   - Surrogate Decision-Making: If the patient is incapacitated and has not expressed their wishes, a legally appointed surrogate (usually a family member or legally designated healthcare proxy) should make decisions in alignment with what the patient would have wanted.

2. Beneficence and Non-maleficence
   - Quality of Life: Evaluating the potential quality of life post-surgery is essential. If the prognosis suggests that the patient will have a significantly diminished quality of life with

severe disability, it may be argued that continued life support might cause more harm than benefit.

- Medical Futility: If the medical team determines that continued life support will not improve the patient's condition or lead to recovery, it may be considered medically futile, justifying the withdrawal of life support.

3. Best Interests

- Holistic Assessment: Consider the overall well-being of the patient, including physical, emotional, and psychological aspects. This includes assessing the burden of continued treatment versus the potential benefits.
- Pain and Suffering: Ensuring that the patient is not subjected to unnecessary pain and suffering is a key ethical consideration.

4. Justice

- Resource Allocation: While resource allocation should not be the primary factor, it can be ethically relevant. Ensuring that resources are used in a manner that benefits the greatest number of patients can be a consideration, especially in resource-limited settings.

5. Family and Social Considerations

- Family Impact: The emotional and financial impact on the family and caregivers should be considered. Supporting the family through the decision-making process is important.
- Cultural and Religious Beliefs: Respect for the cultural and religious beliefs of the patient and their family should guide the decision-making process.

6. Legal Considerations

- Compliance with Laws: Ensure that the decision to withdraw life support complies with relevant laws and regulations, which can vary by jurisdiction.
- Documentation: Proper documentation of the decision-making process and the rationale for withdrawing life support is essential for legal and ethical transparency.

7. Interdisciplinary Approach

- Team Consensus: Decisions should ideally be made with input from an interdisciplinary team, including neurologists, neurosurgeons, intensivists, ethicists, and palliative care specialists to ensure a balanced and comprehensive evaluation.
- Communication: Open and honest communication with the patient’s family about prognosis, treatment options, and potential outcomes is crucial to ensure informed decision-making.

8. Prognostication and Uncertainty

- Prognostic Uncertainty: Recognizing the inherent uncertainties in predicting outcomes after decompressive hemicraniectomy. Decisions should be re-evaluated as new information about the patient's condition becomes available.

In summary, withdrawing life support after decompressive hemicraniectomy for malignant MCA stroke requires a careful and compassionate approach, balancing respect for the patient's autonomy and wishes, the medical assessment of potential outcomes and quality of life, and the ethical principles of beneficence, non-maleficence, justice, and respect for family and cultural values.

23. What does rehabilitation consist of after decompressive hemicraniectomy for malignant MCA stroke and how long will it take?

Rehabilitation after decompressive hemicraniectomy for malignant MCA (middle cerebral artery) stroke is a multifaceted process that involves various stages and therapies aimed at

optimizing recovery and functionality. The specifics of the rehabilitation process and the duration can vary greatly depending on the severity of the stroke, the patient's overall health, and the presence of any complications. Here is an overview of what rehabilitation typically involves and its expected timeline:

Stages of Rehabilitation

1. Acute Phase (1-3 weeks post-surgery)
   - Initial Assessment: Comprehensive evaluation by a multidisciplinary team including neurologists, physiatrists, physical therapists, occupational therapists, and speech-language pathologists.
   - Early Mobilization: Gentle physical activity and mobilization as soon as the patient is medically stable to prevent complications like deep vein thrombosis and pneumonia.
   - Basic Therapies: Initiation of basic physical, occupational, and speech therapy to address immediate deficits and prevent further deterioration.

2. Subacute Phase (3 weeks to 3 months post-surgery)
   - Intensive Therapy: More intensive and structured rehabilitation programs, often in an inpatient rehabilitation facility. This includes:
     - Physical Therapy: Focus on improving motor functions, strength, balance, and coordination. Techniques may include gait training, exercises, and use of assistive devices.
     - Occupational Therapy: Aimed at improving activities of daily living (ADLs) such as dressing, bathing, eating, and writing. Adaptive techniques and equipment may be introduced.
     - Speech and Language Therapy: For patients with aphasia or dysarthria, speech therapy focuses on improving communication skills. Swallowing therapy may also be needed.
     - Cognitive Rehabilitation: For cognitive impairments, therapy might include exercises to improve memory, attention, problem-solving, and executive functions.

3. Chronic Phase (3 months and beyond)
   - Continued Therapy: Depending on progress, ongoing outpatient therapy may be necessary. The intensity and frequency of sessions are tailored to the patient's needs and goals.
   - Community Reintegration: Helping the patient return to community activities, work, or school. This may involve vocational therapy and social support.
   - Home Exercise Program: Patients are often given exercises to continue at home to maintain and further improve their functional gains.

Duration of Rehabilitation

- Initial Hospital Stay: Typically lasts from 1 to 3 weeks post-surgery, during which early rehabilitation efforts begin.
- Inpatient Rehabilitation: Usually lasts 2 to 6 weeks, depending on the patient's progress and insurance coverage.
- Outpatient Rehabilitation: Can continue for several months to years, with periodic reassessments to adjust the therapy plan.

Factors Influencing Recovery

- Stroke Severity: More severe strokes generally require longer and more intensive rehabilitation.
- Age and General Health: Younger patients and those in better pre-stroke health often recover more quickly.

- Complications: The presence of complications, such as infections or additional strokes, can prolong the recovery process.
- Support System: A strong support system involving family, friends, and community resources can significantly enhance recovery outcomes.

Long-Term Outlook

- Functional Outcomes: Many patients make significant improvements in their functional abilities, although some degree of disability may persist.
- Adaptation and Compensation: Learning to adapt to residual deficits and compensating for lost functions are key aspects of long-term rehabilitation.

Each patient's rehabilitation journey is unique, and the approach must be personalized to meet their specific needs and goals. Close collaboration between the patient, their family, and the rehabilitation team is crucial for maximizing recovery potential.

24. How can family members support the patient’s rehabilitation at home after decompressive hemicraniectomy for malignant MCA stroke?

Supporting a patient's rehabilitation at home after a decompressive hemicraniectomy for a malignant middle cerebral artery (MCA) stroke involves a multi-faceted approach. Family members play a crucial role in the recovery process by providing emotional, physical, and practical support. Here are several strategies to effectively support the patient's rehabilitation:

1. Understand the Recovery Process

- Educate Yourself: Learn about the condition, the surgery, and the typical recovery trajectory. This knowledge helps in setting realistic expectations and providing appropriate support.
- Consult Healthcare Professionals: Regularly communicate with the patient's healthcare team to understand their specific needs and progress.

2. Create a Safe and Comfortable Environment

- Home Modifications: Make necessary modifications to the home to accommodate mobility challenges. This might include installing handrails, ramps, and ensuring that frequently used items are easily accessible.
- Safe Space: Arrange furniture and remove any potential hazards to prevent falls and injuries.

3. Assist with Daily Activities

- Personal Care: Help with activities of daily living (ADLs) such as bathing, dressing, and grooming, while encouraging as much independence as possible.
- Mobility Support: Assist with walking, transferring from bed to chair, and using assistive devices like walkers or wheelchairs.

4. Support Rehabilitation Exercises

- Physical Therapy: Encourage and assist with prescribed physical therapy exercises. Follow the therapist's guidelines to ensure exercises are performed correctly and safely.
- Cognitive Exercises: Engage the patient in cognitive activities such as puzzles, memory games, and reading to help with cognitive recovery.

5. Provide Emotional Support

- Positive Environment: Maintain a positive and encouraging atmosphere. Celebrate small victories and progress.

- Active Listening: Be patient and listen to the patient’s concerns and feelings. Emotional support is crucial for mental well-being.

6. Promote Social Interaction

- Family Time: Spend quality time with the patient, involving them in family activities and conversations.
- Social Activities: Encourage participation in social activities, either virtually or in-person, as appropriate, to prevent isolation.

7. Ensure Proper Nutrition and Hydration

- Balanced Diet: Prepare nutritious meals that align with the patient’s dietary needs, ensuring they get adequate protein, vitamins, and minerals.
- Hydration: Monitor and encourage regular fluid intake to prevent dehydration.

8. Medication Management

- Organize Medications: Keep track of medications, dosages, and schedules. Use pill organizers or medication management apps if necessary.
- Monitor Side Effects: Be vigilant about any side effects or adverse reactions and report them to healthcare providers.

9. Regular Medical Follow-ups

- Appointments: Ensure the patient attends all follow-up appointments with their healthcare providers.
- Medical Records: Keep detailed records of the patient’s medical history, medications, and progress to share with healthcare professionals.

10. Seek Professional Help When Needed

- In-home Services: Utilize in-home healthcare services such as visiting nurses, occupational therapists, and speech therapists if recommended.
- Support Groups: Join support groups for stroke survivors and their families to share experiences and gain additional support.

11. Self-Care for Caregivers

- Take Breaks: Caregiving can be demanding. Ensure you take regular breaks to rest and recharge.
- Seek Support: Don’t hesitate to ask for help from other family members, friends, or professional respite care services.

By adopting these strategies, family members can create a supportive and conducive environment for the patient's rehabilitation, promoting better recovery outcomes and improving their quality of life.

25. Are there any new or emerging rehabilitation techniques that could benefit the patient after decompressive hemicraniectomy for malignant MCA stroke?

Decompressive hemicraniectomy is a life-saving surgical procedure for patients with malignant middle cerebral artery (MCA) stroke. Rehabilitation following this surgery is crucial for improving patient outcomes and quality of life. Recent advancements and emerging techniques in post-stroke rehabilitation can offer significant benefits. Here are some of the latest and promising approaches:

1. Virtual Reality (VR) and Augmented Reality (AR) Rehabilitation:

   - VR and AR provide immersive environments that can enhance motor and cognitive rehabilitation. They allow patients to engage in interactive exercises that can improve upper limb function, balance, and spatial awareness.

2. Robotic-Assisted Therapy:
- Robotics can aid in repetitive, high-intensity, and precise movements, which are essential for motor recovery. Devices like exoskeletons and robotic arms help patients practice movements with assistance or resistance tailored to their specific needs.

3. Non-Invasive Brain Stimulation:
- Techniques such as Transcranial Magnetic Stimulation (TMS) and Transcranial Direct Current Stimulation (tDCS) have shown promise in promoting neuroplasticity and improving motor and cognitive function post-stroke. These methods can modulate brain activity and enhance the effects of traditional rehabilitation exercises.

4. Constraint-Induced Movement Therapy (CIMT):
- CIMT involves restricting the use of the unaffected limb, forcing the patient to use the affected limb, thereby improving its function. Modified versions of CIMT are being explored to make the therapy more adaptable and feasible for a broader range of patients.

5. Mirror Therapy:
- This technique uses a mirror to create a reflection of the unaffected limb, giving the illusion that the affected limb is moving. This visual feedback can help improve motor function and reduce pain and neglect in the affected limb.

6. Functional Electrical Stimulation (FES):
- FES involves the application of electrical currents to stimulate muscle contractions in a coordinated manner. It can be used to improve muscle strength, coordination, and functional use of the affected limbs.

7. Task-Oriented and Intensive Practice:
- Rehabilitation programs focusing on task-specific training and high-intensity, repetitive practice have shown better outcomes. Activities of daily living and meaningful tasks are integrated into the therapy to enhance motor recovery and functional independence.

8. Neurofeedback and Brain-Computer Interfaces (BCIs):
- These technologies allow patients to receive real-time feedback on their brain activity and control external devices using their brain signals. BCIs, in particular, can help in motor rehabilitation by enabling patients to practice movement through mental imagery and intention.

9. Tele-rehabilitation:
- Tele-rehabilitation leverages telecommunication technologies to provide remote rehabilitation services. This approach ensures continuity of care, especially for patients with limited access to rehabilitation centers, and allows for regular monitoring and adjustments to the rehabilitation program.

10. Pharmacological Interventions:
- Research is ongoing into drugs that can enhance neuroplasticity and recovery. Medications that target neurotransmitter systems, neurotrophic factors, and inflammatory pathways are being explored as adjuncts to traditional rehabilitation.

Implementing these advanced techniques requires a multidisciplinary approach, including neurologists, physiotherapists, occupational therapists, speech therapists, and neuropsychologists. Tailoring the rehabilitation program to the specific needs and abilities of each patient is crucial for optimizing recovery after a decompressive hemicraniectomy for malignant MCA stroke.
